# The Key Regulator of Necroptosis, RIP1 Kinase, Contributes to the Formation of Astrogliosis and Glial Scar in Ischemic Stroke

**DOI:** 10.1007/s12975-021-00888-3

**Published:** 2021-02-24

**Authors:** Yong-Ming Zhu, Liang Lin, Chao Wei, Yi Guo, Yuan Qin, Zhong-Sheng Li, Thomas A. Kent, Claire E. McCoy, Zhan-Xiang Wang, Yong Ni, Xian-Yong Zhou, Hui-Ling Zhang

**Affiliations:** 1 Jiangsu Key Laboratory of Neuropsychiatric Diseases and College of Pharmaceutical Sciences, Laboratory of Cerebrovascular Pharmacology, College of Pharmaceutical Science, Jiangsu Key Laboratory of Preventive and Translational Medicine for Geriatric Diseases, School of Public Health, Soochow University, 199 Ren-Ai Road, Suzhou, 215123 Jiangsu China; 2 The First Affiliated Hospital of Xiamen University, Xiamen, 361001 Fujian China; 3 Department of Cardiology, The First Affiliated Hospital of Soochow University, 188 Shi-Zi Road, Suzhou, 215006 Jiangsu China; 4 Institute of Biosciences and Technology, Texas A&M Health Science Center, Department of Neurology, Houston Methodist Hospital, Houston, TX USA; 5 School of Pharmacy and Biomolecular Sciences, Royal College of Surgeons in Ireland, 123 St Stephens Greens, Dublin 2, Ireland

**Keywords:** Ischemic stroke, RIP1 kinase, Astrocyte, Glial scar, VEGF-D, VEGFR-3

## Abstract

Necroptosis initiation relies on the receptor-interacting protein 1 kinase (RIP1K). We recently reported that genetic and pharmacological inhibition of RIP1K produces protection against ischemic stroke-induced astrocytic injury. However, the role of RIP1K in ischemic stroke-induced formation of astrogliosis and glial scar remains unknown. Here, in a transient middle cerebral artery occlusion (tMCAO) rat model and an oxygen and glucose deprivation and reoxygenation (OGD/Re)-induced astrocytic injury model, we show that RIP1K was significantly elevated in the reactive astrocytes. Knockdown of RIP1K or delayed administration of RIP1K inhibitor Nec-1 down-regulated the glial scar markers, improved ischemic stroke-induced necrotic morphology and neurologic deficits, and reduced the volume of brain atrophy. Moreover, knockdown of RIP1K attenuated astrocytic cell death and proliferation and promoted neuronal axonal generation in a neuron and astrocyte co-culture system. Both vascular endothelial growth factor D (VEGF-D) and its receptor VEGFR-3 were elevated in the reactive astrocytes; simultaneously, VEGF-D was increased in the medium of astrocytes exposed to OGD/Re. Knockdown of RIP1K down-regulated VEGF-D gene and protein levels in the reactive astrocytes. Treatment with 400 ng/ml recombinant VEGF-D induced the formation of glial scar; conversely, the inhibitor of VEGFR-3 suppressed OGD/Re-induced glial scar formation. RIP3K and MLKL may be involved in glial scar formation. Taken together, these results suggest that RIP1K participates in the formation of astrogliosis and glial scar via impairment of normal astrocyte responses and enhancing the astrocytic VEGF-D/VEGFR-3 signaling pathways. Inhibition of RIP1K promotes the brain functional recovery partially via suppressing the formation of astrogliosis and glial scar.

**Graphical Abstract:**

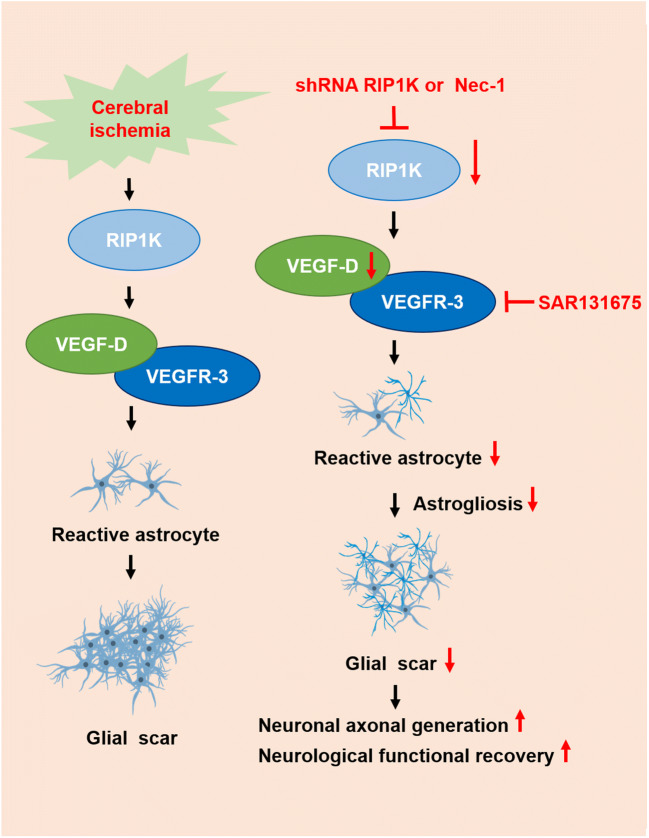

**Supplementary Information:**

The online version contains supplementary material available at 10.1007/s12975-021-00888-3.

## Introduction

Stroke remains one of the most devastating diseases although stroke incidence and sequelae have now been improved due to a combination of stroke prevention and treatment strategies [1, 2]. Tissue-type plasminogen activator (tPA) and endovascular therapy use may play a role in improved outcomes, but there remain delays in contacting emergency medical services reducing opportunities for tPA treatment due to a narrow time window of up to 4.5 h after symptom onset [3, 4] and endovascular intervention is not universally available. Thus, identifying novel ways to treat stroke continues to be a high priority.

As the most abundant cell population in the brain, astrocytes play multiple critical roles in supporting brain physiological function, including maintenance of ionic and osmotic homeostasis, regulation of metabolism of major neurotransmitters, remodeling extracellular space, coupling neurovascular functions and regulating blood–brain barrier (BBB) integrity, and providing inflammatory defense and anti-oxidant activity [5–7]. Astrocytes respond to ischemic stroke damage by a hallmark process of reactive astrogliosis and subsequent glial scar formation, characterized as hypertrophic morphology and over-proliferation of astrocytes and increased expression of the intermediate filament protein, glial fibrillary acidic protein (GFAP) [8–11]. Reactive astrocytes are the main cells consisting the glial scar, although oligodendrocytes and microglia are also included [12].

Accumulating evidence indicates that astrogliosis performs both beneficial and/or detrimental functions depending on the brain injury time and context [13, 14]. In the acute phase following ischemic damage, reactive astrocytes are beneficial for tissue repair via secretion of anti-inflammatory factors, uptake of excess glutamate, and isolation of undamaged tissue [15–17]. Concomitantly, reactive astrocytes are known to exacerbate injury via the release of neurotoxic levels of reactive oxygen species, amplify inflammation via cytokine production, compromise BBB function via VEGF production, and aggravate cytotoxic edema through stimulation of AQP4 [18–22]. More importantly, in the chronic phase, about 2 to 4 weeks after ischemic stroke, a glial scar, a physical and functional wall, is formed around the necrotic brain tissue of the infarct. In the glial scar, reactive astrocytes express a broad range of inhibitory molecules against axonal regeneration, such as chondroitin sulfate proteoglycans (CSPGs) including neurocan and phosphacan, which have been recognized as major barriers to central nervous system (CNS) axon extension and thus regeneration failure in the CNS [2, 23–26]. Therefore, the complex aspects of astrogliosis function drive profound interest in developing a better understanding of the distinct molecular and relevant signaling cascades regulating reactive astrogliosis and, in particular, in searching for targets that promote tissue functional recovery for stroke patients [27–30].

In recent years, a highly regulated form of necrosis, termed regulated necrosis or necroptosis, has been discovered and elicited a significant interest in studying the implication of necroptosis in human diseases including ischemic stroke [31]. Necroptosis is a form of cell death similar to necrosis in terms of morphological features, but it can be regulated in a caspase-independent manner [32]. Receptor-interacting protein 1 kinase (RIP1K) is a crucial mediator of necroptosis [31, 33]. The activation of RIP1K leads to the recruitment of RIP3K, forming the RIP1-RIP3 complex; subsequently, the kinase of RIPK3 phosphorylates mixed lineage kinase domain-like protein (MLKL) causing MLKL to form a pore-like structure, resulting in rapid plasma membrane lysis—a hallmark of necrotic cell death [34–38].

Necrostatin (Nec-1) can specifically inhibit the activity of RIP1K via preventing RIP1-RIP3 interaction. Necroptosis can be inhibited with Nec-1 in many kinds of CNS disease, such as ischemia, brain injury, and intracerebral hemorrhage [39]. However, almost all studies have been performed from the viewpoint of neurons. More recently, we have found that RIP1K and RIP3K were up-regulated in the ischemic cortex following ischemic stroke and pharmacological or genetic inhibition of RIP1K conferred neuroprotection against ischemic stroke partly associated with its protection on ischemic astrocytes [31].

In the brain, VEGF mediates angiogenesis, neural migration, and neuroprotection. However, excessive VEGF disrupts intracellular barriers, increases leakage of the choroid plexus endothelia, evokes edema, and activates the inflammatory pathway [40]. The VEGF family includes seven different homologous members, including VEGF-A, VEGF-B, VEGF-C, VEGF-D, VEGF-E, VEGF-F, and placental growth factor [41]. VEGF-D exerts its function through high affinity tyrosine kinase VEGFR-3. VEGFR-3 in the peri-infarction penumbra region is predominantly expressed in reactive astrocytes, suggesting that VEGFR-3 may be involved in the glial reaction during ischemic insults [41].

Our preliminary experimental results unexpectedly found that knockdown of RIP1K in astrocytes markedly down-regulated the VEGF-D gene *Figf* with microarray analysis. The goal of this study was to test the hypothesis that RIP1K may be involved in ischemic stroke-induced formation of astrogliosis and glial scar via regulating the VEGF-D/VEGFR-3 signaling pathways in astrocytes.

## Materials and Methods

### Animals

Male Sprague-Dawley (SD) rats (aged 8 ~ 9 weeks, weight 280 g to 320 g, fasting blood glucose, 4 ~ 5.5 mmol/l) were purchased from SLAC Company (Shanghai, China). All animal procedures and protocols used in this study were approved by the Soochow University Animal Care and Use Committee (use license: SYXK-2016-0050; production license: SYXK-2017-0006). All animals were fed standard diet and were kept under 12 h cycles of light and darkness. All procedures were designed to minimize both the suffering and the number of animals used.

**Fig. 1:**
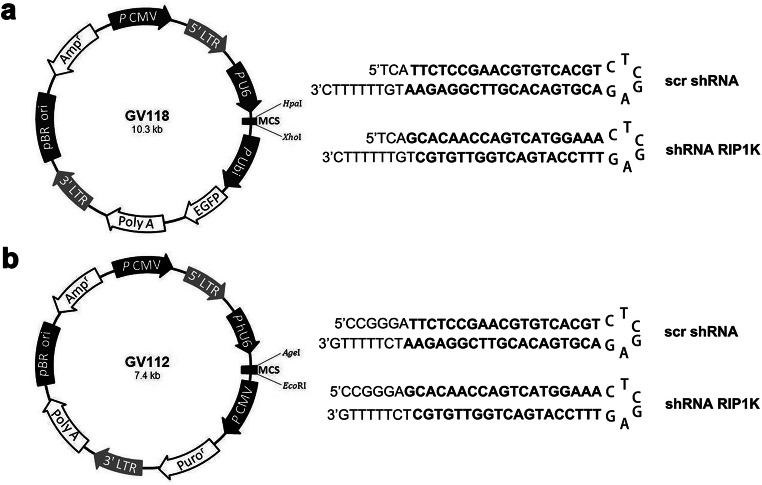
The schematic illustration of the shRNA-expressing lentiviral constructs. **a** The designed lentivirus for RIP1K with EGFP construct contained a unique 19-nt sense and antisense strands, a loop sequence (5′-CTCGAG-3′), the RNA PloIII terminator (5′-TTTTTTC-3′), and 5′ single-stranded overhangs for ligation into HpaI- and XhoI-digested lentivirus vector. **b** The designed lentivirus for RIP1K without EGFP construct contained a unique 19-nt sense and antisense strands, a loop sequence (5′-CTCGAG-3′), the RNA PloIII terminator (5′-TTTTTG-3′), and 5′ single-stranded overhangs for ligation into AgeI- and EcoRI-digested lentivirus vector

To induce RIP1K knockdown in rat’s brain, rats received intracerebroventricular injections of lentiviruses with shRNA RIP1K (short hairpin RNA targeting rat RIP1K) or with control scrambled shRNA (scr shRNA), produced by GeneChem Co., Ltd. (Shanghai, China). Rats were subjected to transient focal cerebral ischemia 5 days after shRNA RIP1K treatments. We have two types of lentivirus with or without an enhanced green fluorescent protein (EGFP) (Fig. 1).

The target sequence for RIP1K is as follows: shRNA RIP1K: 5′-GCAGTTCTTGGTCTGCATA-3′; scr shRNA: 5′-TTCTCCGAACGTGTCACGT-3′.

**Fig. 2:**
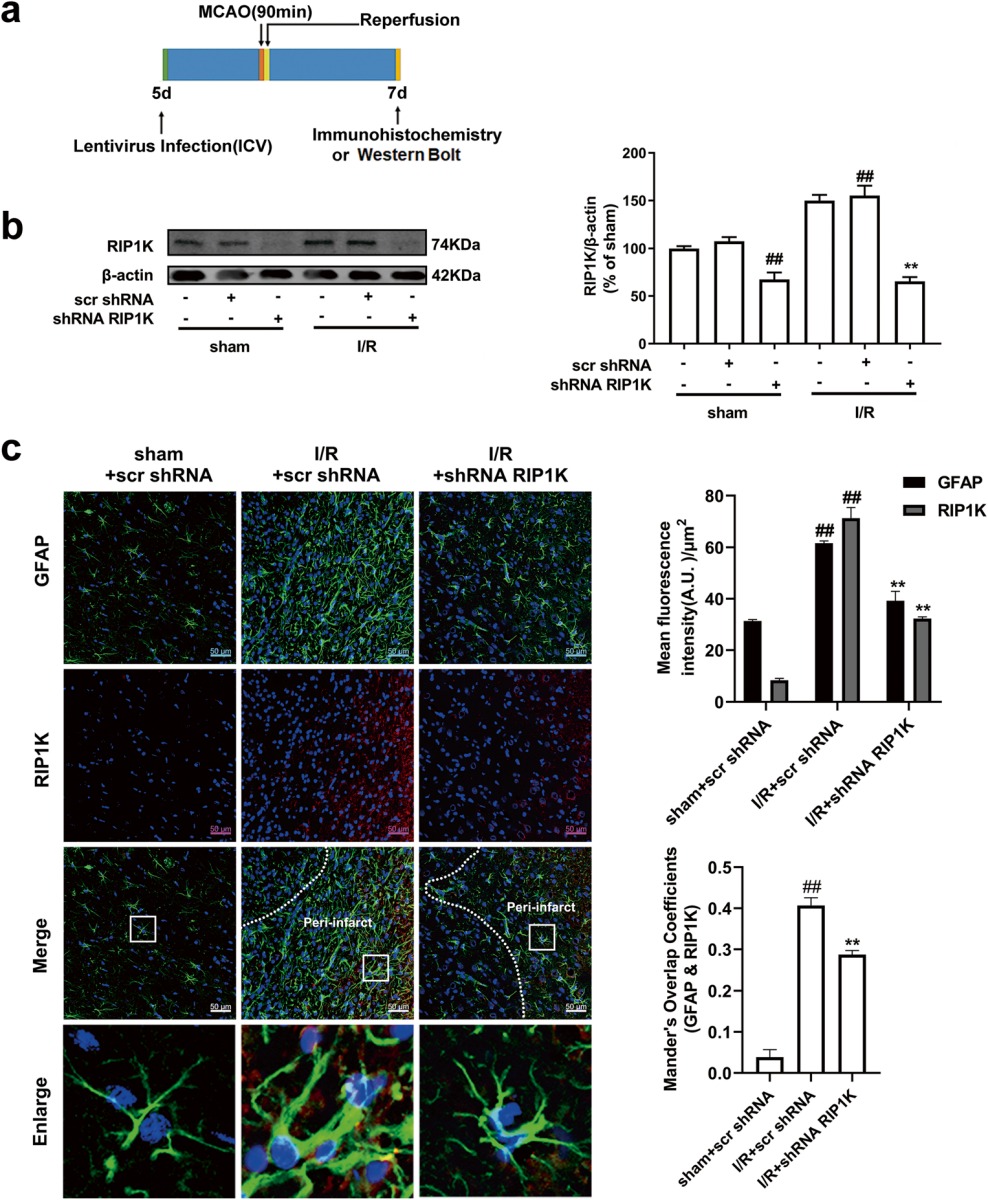
Knockdown of RIP1K down-regulates RIP1K level of cerebral cortex. **a** Experimental protocol. **b** Western blotting analysis indicates that shRNA RIP1K treatment down-regulates RIP1K level of cerebral cortex both in rats at day 7 after I/R and in sham-operated rats. Data are mean ± SD, *n* = 3. ^##^*P* < 0.01 vs. sham + scr shRNA group; ^**^P < 0.01 vs. I/R + scr shRNA group. **c** Knockdown of RIP1K reduces the protein level of RIP1K in astrocytes. Representative images of cerebral cortex double staining for GFAP (green) and RIP1K (red) in rats 7 days after I/R or in sham-operated rats. Hoechst (blue) was used to stain the nucleus. The white dotted line represents the edge between the infarct area and the peri-infarct area, and the white boxes indicate the corresponding area of the enlarged images shown below. Mander’s overlap coefficient demonstrated the colocalization between GFAP and RIP1K. Data are mean ± SD, *n* = 3. ^##^*P* < 0.01 vs. sham + scr shRNA group; ^**^P < 0.01 vs. I/R + scr shRNA group

Western blotting analysis confirmed that the RIP1K gene was successfully silenced in the brain (Fig. 2b) or in cultured primary astrocytes (Fig. 6a). Immunohistochemistry results showed that RIP1K was knocked down in astrocytes after lentivirus treatment (Fig. 2c).

### Transient Middle Cerebral Artery Occlusion (MCAO) Model

The right middle cerebral artery (MCA) occlusion (MCAO) for 90 min and reperfusion model was induced as previously described [42]. Briefly, with anesthetic treatment, a rubber silicon-coated 4-0 monofilament suture was inserted into the right internal carotid artery via the common carotid artery and was advanced until the tip of the monofilament occluded the origin of the right MCA. Study inclusion criteria of diminished (>80%) and reperfused (80% of baseline) blood flow were detected with Laser-Doppler flowmetry (moorVMS) [26]. Animals were excluded from the study if the hemorrhage occurred during the procedure or they died during or following surgery. Surgical sham controls were performed without the occlusion of the MCA. The rectal temperature was maintained at 37 ± 0.5 °C using a heating pad throughout the entire procedure.

#### Necrostatin-1 (Nec-1) Treatment and Injection

To observe the effect of delayed administration of Nec-1 on the glial scar markers or the thickness of glial scar, the rats were randomly assigned to groups by using the online tool Quickcalcs (http://www.graphpad.com/quickcalcs/). Nec-1 at 24 nmol was injected intracerebroventricularly (icv) stereotaxically at the coordinates of 1.5 mm posterior to the bregma, 1.5 mm lateral from the midline, and 4.0 mm depth to the cortical surface above the lateral ventricles, starting from 24 h post-tMCAO through a stainless steel guide cannula for 7 days or through an osmotic pump system for 14 days.

To observe the effect of long-term use of Nec-1 on the formation of glial scar, intracerebroventricular infusion of Nec-1 through an osmotic pump system was started at 24 h after tMCAO for 14 days. The mini-osmotic pump (flow rate 0.56 μl/h; reservoir 200 μl) was attached to the cannula and inserted into a subcutaneous pocket. The incision was closed in two layers with absorbable suture. The cannula and the mini-osmotic pump were filled with Nec-1 solution (17.8 mM). 24 nmol Nec-1 was intracerebroventricularly administered before the mini-osmotic pump system worked.

### Assessment of Neurological Deficits and Brain Atrophy

Behavioral testing took place before tMCAO (baseline) or/and at 1, 3, 7, 14, 21, and 28 days after tMCAO as described previously [43] Neurological deficit score was performed according to a 6-point scale: 0, no neurological deficit performance; 1, ptosis of eyelid ipsilateral to the occluded MCA side and/or failure to stretch ipsilateral forelimb; 2, persistently walks toward the ipsilateral side in large circles; 3, persistently walks toward ipsilateral side in small circles and/or rolls over repeatedly; 4, lies nearly motionless on the contralateral side; and 5, dies after recovery from the anesthesia. Grip strength (force, g) was evaluated with a digital force-gauging apparatus (YLS-13A, Jinan YiYan Technology Development Company Limited, China), ten trials for each animal. For the cylinder test, the rat was placed in a Plexiglas cylinder for 10 min. A total of twenty forelimb movements were recorded. The final score was calculated as follows: (Right forelimb movement − Left forelimb movement) / (Right forelimb movement + Left forelimb movement + Both movements).

Brain atrophy was measured at 28 days after tMCAO; the atrophy volume was measured as follows: (RA = LT − RT, where RA = atrophy volume in the right hemisphere, LT = total cortex volume in the left hemisphere of the same brain, and RT = total cortex volume in the right hemisphere of the same brain).

### Primary Astrocyte Cultures, Oxygen-Glucose Deprivation (OGD)/ Reoxygenation (OGD/Re), and Lactate Dehydrogenase (LDH) Leakage Measurement

#### Primary Astrocyte Cultures

Primary cortical astrocyte cultures were prepared from 24 h postnatal Sprague-Dawley rats as described previously [43]. Briefly, the cerebral cortices were digested with 2.5% trypsin for 10 min at 37 °C and filtered through a sterile 40 μm nylon cell strainer. Cells were cultured in DMEM/F12 (GIBCO, 11330) containing 10% fetal bovine serum (GIBCO, 10099) and 1% 100 U/ml penicillin/streptomycin (Beyotime, C0222). The cultures were maintained at 37 °C under >90% humidity and 5% CO_2_. The medium was changed every 2 ~ 3 days until cells reached confluence. More than 95% of the cultured cells were astrocytes as identified by immunofluorescence staining for GFAP, an astrocytic marker protein.

#### Oxygen-Glucose Deprivation (OGD)/Reoxygenation (OGD/Re)

For OGD treatment, the astrocytes were washed with 0.01 M phosphate buffer saline (PBS), incubated in serum- and glucose-free DMEM (GIBCO, 11966), placed in a humidified hypoxia chamber (Billups-Rothenberg, CA, USA), and flushed with mixed gas (95% N_2_/5% CO_2_) for 10 min to remove the oxygen and then incubated in the sealed hypoxia chamber (Billups-Rothenberg, CA, USA) for 6 h at 37 °C. For OGD/Re, cells were taken out of the chamber following OGD, transferred to the regular cell culture incubator, and incubated with complete medium. Control cells were incubated in complete medium and received similar wash steps as OGD/Re-treated cells in a humidified atmosphere with 5% CO_2_ at 37 °C. To induce RIP1K knockdown in astrocytes, lentiviruses with shRNA RIP1K or with scr shRNA were added to the 3rd passage of the astrocytes. Nec-1 (Selleck, S8037) at 100 μM, GSK′872 (MCE, HY-101872) at 10 μM, Necrosulfonamide (NSA) (MCE, HY-100573) at 1 μM, or SAR131675 (MCE, HY-15458) at 20 nM was added to the cells upon reoxygenation.

#### Lactate Dehydrogenase (LDH) Leakage Measurement

To determine cell injury, the lactate dehydrogenase (LDH) leakage from astrocytes was detected using an LDH assay kit (Nanjing Jiancheng, A020) at 450 nm using a multimode microplate reader, Infinite® M1000 PRO (Tecan Trading AG, Switzerland), as previously described [42].

### Astrocyte and Neuron Co-Culture and Neurite Growth Quantification

Astrocyte and neuron co-culture: astrocytes were seeded into Petri dishes and exposed to OGD for 6 h following reoxygenation for 24 h, and then, neurons were seeded into the Petri dishes and incubated together with astrocytes for additional 24 h. In this method, neurons and astrocytes have direct contact. All the Petri dishes were coated previously with poly-D-lysine (0.1 mg/ml). For the control group, neurons were co-cultured with astrocytes which were not exposed to OGD. Then, cells were stained with double immunofluorescence including a neuronal marker β-III-tubulin and an astrocytic marker GFAP and neurite growth was quantified.

#### Primary Cortical Neuron Culture

Primary cortical neurons were prepared from embryonic day 18 Sprague-Dawley rats as described previously with the following modifications [44]. In brief, the cerebral cortices were digested with 2.5% trypsin for about 10 min at 37 °C and filtered with a sterile 40 μm nylon cell strainer, and then, neurons were seeded into the Petri dishes and incubated together with astrocytes in co-culture medium (neurobasal medium (GIBCO, 21103-049) : DMEM/F12 (GIBCO, 11330), 1 : 1) supplemented with 2% B-27 (GIBCO, 17504-044), 10% fetal bovine serum (GIBCO, 10099), 0.5 mM L-Glutamine (Sigma, G8540), and 1%100 U/ml penicillin/streptomycin (Beyotime, C0222) in a humidified CO_2_ incubator (5% CO_2_, 37 °C) for 24 h.

#### Immunofluorescence

The cells were rinsed with PBS three times and fixed in 4% paraformaldehyde for 10 min. Then, cells were incubated for 30 min in 0.3%Triton X-100 and rinsed three times with PBS, followed by bovine serum albumin for 1 h. The cells were incubated overnight at 4 °C with glial fibrillary acid protein (GFAP) antibody (1 : 500, Abcam, ab7260) and β-III-tubulin antibody (1 : 500, Abcam, ab78078). Then, cells were incubated with secondary antibodies (Supplementary Table S2) for 1 h at room temperature, followed by Hoechst (1 : 10,000, Sigma, 33258) solution to stain nuclei for 20 min. Images were obtained by a confocal laser scanning microscopy (LSM 710, Carl Zeiss, Germany).

#### Neurite Growth Quantification

Neurite growth was quantified as previously described [44]. Briefly, every neurite for each neuron on the glass coverslips was measured by FIJI ImageJ software using neuronJ plugins and the longest length of neurite for each neuron was recorded. And then, ten neurons with the longest neurites in each glass coverslips were selected to calculate their average neurites.

### Proliferation Assay

Astrocyte proliferation in vitro was determined by the Cell-Light EdU Apollo®567 In Vitro Kit (Riobio, C10310) following the manufacturer’s instructions. Astrocytes were incubated with 20 μM EdU for 24 h after OGD/Re. Next, 4% paraformaldehyde was used to fix the cell morphology for 30 min at room temperature and the cells were permeabilized with 0.5% Triton X-100 for 10 min. Finally, the Apollo®567 reaction solution was added to the cells and the cells were incubated for 30 min. The cells were incubated overnight with an anti-GFAP antibody (1 : 1000, Abcam, ab10062). Nuclei were stained with Hoechst (1 : 5000, Sigma, 33258), and images were obtained by a confocal microscope (LSM 710; Carl Zeiss Co. Ltd., Oberkochen, Germany).

### Immunohistochemistry

Immunohistochemistry was performed as previously described [45]. Brain sections were fixed with 4% paraformaldehyde for 10 min, permeabilized with 0.3% Triton X-100 and blocked by 1% BSA for 1 h at room temperature, and incubated with specific primary antibodies (Supplementary Table S1) overnight at 4 °C and corresponding secondary antibodies (Supplementary Table S2) for 1 h at room temperature. Then, the sections were incubated for 20 min with Hoechst (1 : 10,000, Sigma, 33258) solution to stain nuclei. Images were obtained by a confocal laser scanning microscopy (LSM 710, Carl Zeiss, Germany) or a fluorescence microscope (IX73, Olympus, Japan). For rats with ischemic stroke, we could distinguish the infarct core and the peri-infarct zone, and then, we measured the fluorescence intensity of the peri-infarct zone where astrogliosis and glial scar were formed. For sham-operated rats, the fluorescence intensity was determined over the entire image in the corresponding area of cerebral cortex. Fluorescence intensity was determined using built-in and custom-written ImageJ plugins and normalized to the background. Results are expressed as mean fluorescence intensity (fluorescence intensity per unit area, A.U./μm^2^). Colocalization analysis for double staining images was measured by FIJI ImageJ software using colocalization finder. Mander’s overlap coefficients indicate an actual overlap between color channels, which represents the true level of colocalization. Values range from 0 to 1, and the results are 1 for perfect overlap and 0 for no overlap. Mander’s overlap coefficients are suitable when the fluorescence of one antigen is stronger than another antigen.

### Western Blotting Analysis

The proteins extracted from ischemic cortex and cultured astrocytes were separated with 6% ~ 10% SDS-PAGE gel and transferred to nitrocellulose membranes and then blocked with 5% non-fat milk for 1 h. The transferred membranes were incubated with specific primary antibodies (Supplementary Table S1) overnight at 4 °C and corresponding secondary antibodies (Supplementary Table S2) for 1 h at room temperature. Immunoreactivity was detected with an Odyssey scanner (LI-COR). Blots were captured by the Odyssey scanner (LI-COR). Densitometric analysis of the bands was quantitatively analyzed with FIJI Image J. *β*-Actin (1 : 5000, Sigma, A5441) served as the loading control.

### Enzyme-Linked Immunosorbent Assay (ELISA)

The astrocyte medium was centrifuged for 5 min, 5000 *g*, at 4 °C to remove cell debris. VEGF-D levels in the astrocyte medium were analyzed with rat’s VEGF-D ELISA kit (Sinobestbio China) following the manufacturer’s instructions, and the results are presented as ng/ml or ng/mg. Absorbance was measured at 450 nm using a multimode microplate reader, Infinite® M1000 PRO (Tecan Trading AG, Switzerland).

### Rat Recombinant VEGF-D-Induced Formation of Glial Scar

When astrocytes grew to 70% ~ 80% convergence, the astrocytes were treated with rat recombinant VEGF-D (400 ng/ml, Sino Biological, 80104-R01H) for 48 h, and then, the cells were harvested and the glial scar markers were detected with western blotting analysis.

### Brain Histology

The forebrains were divided into five coronal sections (3 mm/each) using a rat brain matrix (Harvard apparatus). The brain section was fixed in 4% paraformaldehyde in PBS, embedded in paraffin, and cut into 8 *μ*m slices. Slices were deparaffinized and stained with hematoxylin and eosin (H&E). Necroptosis was identified as previously described [46]. Lesion region and necrotic-like cells were identified as previously described [46–48]. The number of necrotic cells was determined in the lesion area of each section by the FIJI ImageJ software.

### Microarray Analysis for Gene Expression

Primary cultured astrocytes were randomly divided into two groups: OGD 6 h + scr shRNA group and OGD 6 h + shRNA RIPK1 group (*n* = 3/group). Cells were harvested and sent to GeneChem Co., Ltd. (Shanghai, China) for gene expression profiling analysis. The data for each group were analyzed using the Cluster 3.0 program (http://bonsai.hgc.jp/~mdehoon/software/cluster/software.htm). A subset of genes with log_2_ |Fold change| ≥ 1 and *P* < 0.05 (from a two-sample *t* test) was selected for clustering analysis.

### Statistical Analysis

The data are presented as mean ± SD. Statistical analysis for multiple comparisons was performed by one-way analysis of variance (ANOVA) with Tukey’s test or two-way ANOVA followed by a post hoc Tukey’s test, and the difference between the two groups was evaluated by unpaired Student’s *t* test. The nonparametric data, such as neurological deficits score, was evaluated by multiple T tests in which statistical significance was determined using the Bonferroni-Dunn method. *P* < 0.05 was considered statistically significant.

## Results

### RIP1K Is Elevated in the Formation of Astrogliosis and Glial Scar Following Ischemia/Reperfusion or OGD/Re In Vivo and In Vitro

**Fig. 3:**
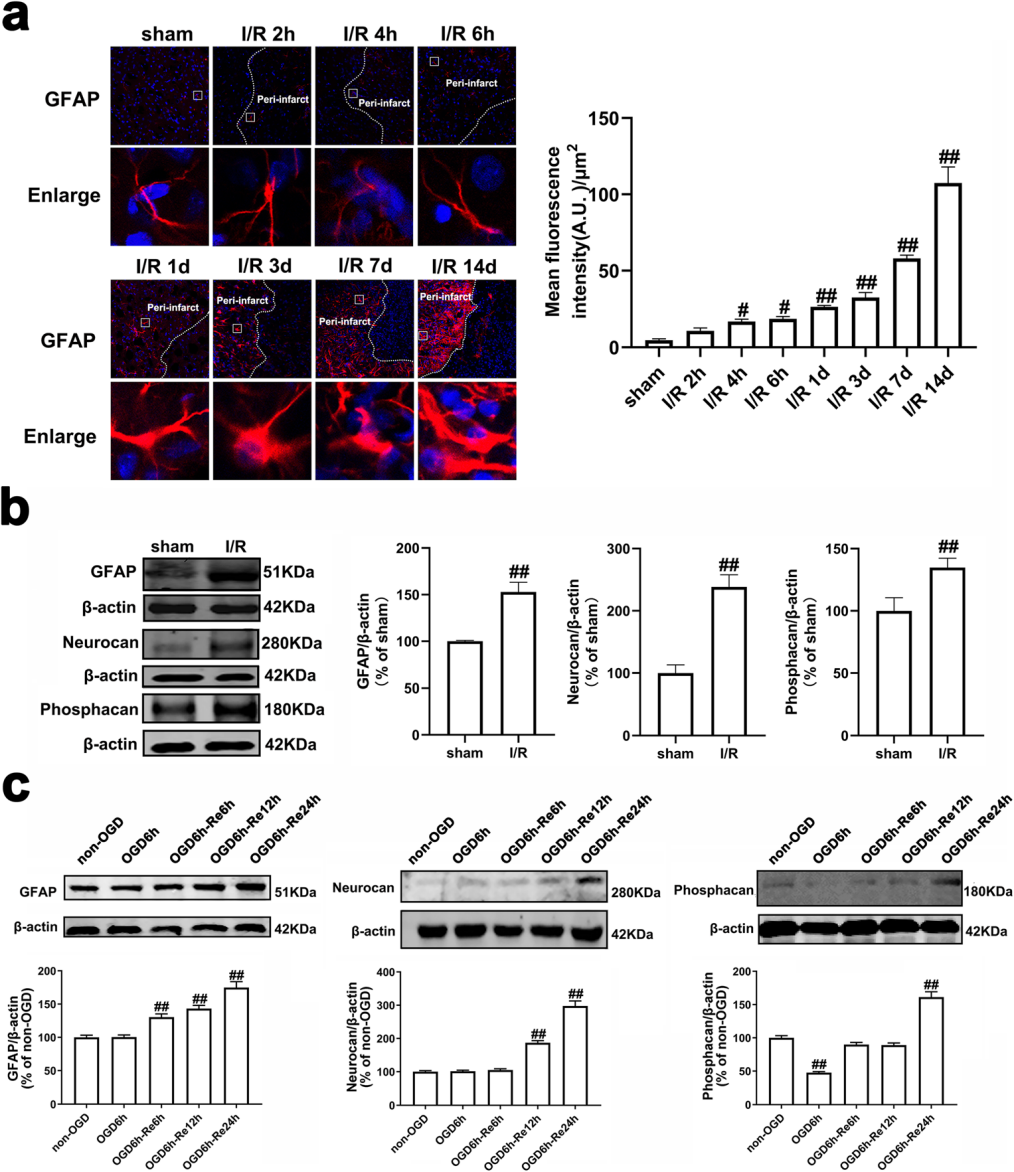
The formation of astrogliosis and glial scar following I/R or OGD/Re in vivo and in vitro. **a** Transient ischemic stroke was induced by MCAO for 90 min followed by reperfusion (I/R). Representative images of GFAP (red) positive astrocytes were shown in the cerebral cortex of rats. Hoechst (blue) was used to stain the nucleus. The white dotted line represents the edge between the infarct area and the peri-infarct area, and the white boxes indicate the corresponding area of the enlarged images shown below. GFAP immunostaining was quantified by using red fluorescence intensity. Data are mean ± SD, *n* = 3. ^##^*P* < 0.01 vs. sham group. **b** Representative images of GFAP, neurocan, and phosphacan in the peri-infarct region at 7 days after I/R with western blotting analysis. Data are mean ± SD, *n* = 3. ^##^*P* < 0.01 vs. sham group. **c** The time-course changes of GFAP, neurocan, and phosphacan levels after OGD/Re injury with western blotting analysis. Astrocytes were exposed to OGD for 6 h and reoxygenation for 6 h, 12 h, and 24 h. Data are mean ± SD, *n* = 3. ^##^*P* < 0.01 vs. non-OGD group. Statistical analysis for multiple comparisons was performed by one-way ANOVA with Tukey’s test by a post hoc Tukey’s test, and the difference between two groups was evaluated by unpaired Student’s *t* test

Astrocytes can become reactive following ischemia. The hallmark of reactive astrogliosis is the morphological changes and the increased expression levels of GFAP [49, 50]. Reactive astrocytes eventually form glial scar in the penumbra that demarcates the ischemic core (infarction) from healthy tissue [51]. Thus, GFAP expression has been used to inspect the morphological changes during astrogliosis following ischemia. We first conducted a detailed study on the morphology of reactive astrocytes in the penumbra of cerebral cortex at 2 h, 4 h, 6 h, 1 d, 3 d, 7 d, and 14 d after reperfusion following MCAO for 90 min based on GFAP staining. We found that there is little expression of GFAP in the cortex of sham-operated rats. No significant differences were found in the GFAP level between the sham-operated group and the I/R 2 h group. From 4 h to 14 d after focal ischemia, the expression of GFAP was significantly up-regulated over time. Methodologically, in the acute phase after focal ischemia (from 1 d to 3 d after focal ischemia), astrocytes exhibited stellate morphology and hypertrophied GFAP-positive processes over time. In the subacute phase after focal ischemia (7 d after focal ischemia), astrocytes exhibit elongated processes pointing to the ischemic core, and a glial scar forms. In the chronic phase after focal ischemia (14 d after focal ischemia), astrocytes further extended processes toward the ischemic core and the glial scar matures during this period (Fig. 3a). Glial scar formation is marked by the deposition of different extracellular matrix (ECM) components (e.g., neurocan and phosphacan) [11, 12]. Then, we determined the expression of GFAP, neurocan, and phosphacan in the peri-infarct region at 7 d after I/R with western blotting analysis. Compared to the sham group, the protein levels of GFAP, neurocan, and phosphacan in the peri-infarct of cerebral cortex were markedly increased at 7 d after I/R injury (Fig. 3b).

To mimic I/R injury in vitro, we established OGD for 6 h followed by reoxygenation (OGD/Re) for 12 h or 24 h astroglial scar formation model in primary cultured astrocytes. Consistent with the literature by Wang and colleagues [29], the results showed that OGD6h/Re induced an increase in the protein levels of GFAP, neurocan, and phosphacan over time and a peak at OGD6h/Re24h (Fig. 3c).

**Fig. 4:**
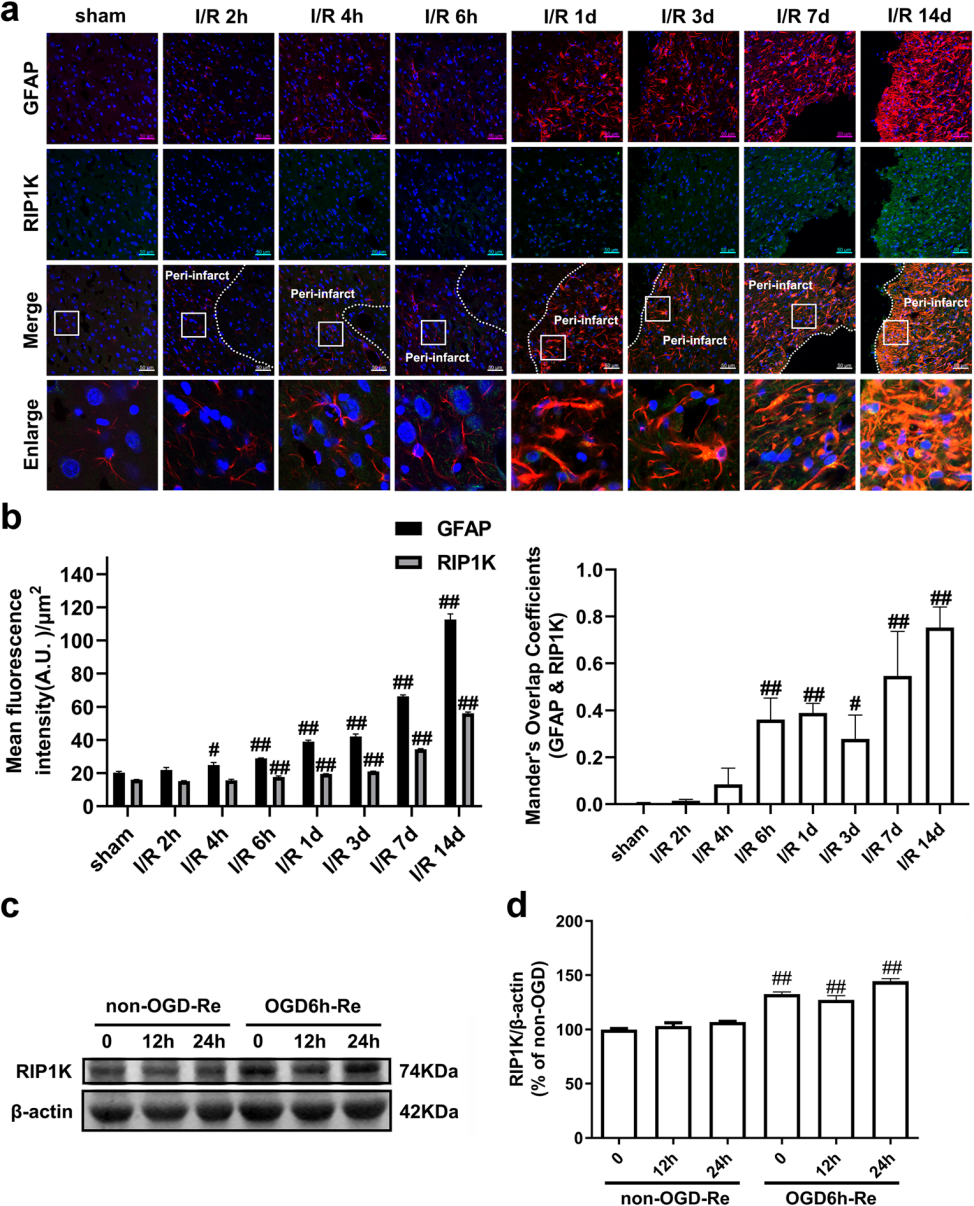
The time-course changes of RIP1K protein level in astrocytes following I/R or OGD/Re. **a** Transient ischemic stroke was induced by MCAO for 90 min followed by reperfusion (I/R). Representative images of double staining for GFAP (red) and RIP1K (green) in the cerebral cortex of rats. Hoechst (blue) was used to stain the nucleus. The white dotted line represents the edge between the infarct area and the peri-infarct area, and the white boxes indicate the corresponding area of the enlarged images shown below. **b** Quantification of fluorescence intensity of RIP1K and GFAP immunostaining in **a**. Mander’s overlap coefficient demonstrated the colocalization between GFAP and RIP1K. Data are mean ± SD, *n* = 3. ^##^*P* < 0.01 vs. sham group. **c**, **d** The time-course changes of RIP1K level in astrocytes after OGD/Re injury with western blotting analysis. Astrocytes were exposed to OGD for 6 h followed by reoxygenation for 12 h or 24 h. Data are mean ± SD, *n* = 3. ^#^*P* < 0.05, ^##^*P* < 0.01 vs. non-OGD group. Statistical analysis was carried out with one-way ANOVA followed by a post hoc Tukey’s test

Previously, we reported that RIP1K protein level was increased in the ischemic brain after permanent middle cerebral artery occlusion and in OGD-induced astrocytic cell injury [31]. It is unknown whether RIP1K protein level is increased in the reactive astrocytes and in the formation of glial scar following tMCAO. The results of double immunohistochemistry revealed that from 6 h to 14 d after focal ischemia, the levels of RIP1K in GFAP-positive astrocytes were significantly up-regulated over time (Fig. 4a, b).

Consistent with the results in vivo, we next found that the protein levels of RIP1K were increased at 6 h post-OGD and peaked at OGD6h/Re24h in the reactive astrocytes (Fig. 4c, d). There was no difference of RIP1K levels between the non-OGD group and the non-OGD/Re12h group or the non-OGD/Re24h group.

### Knockdown of RIP1K Attenuates Ischemic Stroke-Induced Formation of Astrogliosis and Glial Scar In Vivo and In Vitro

**Fig. 5:**
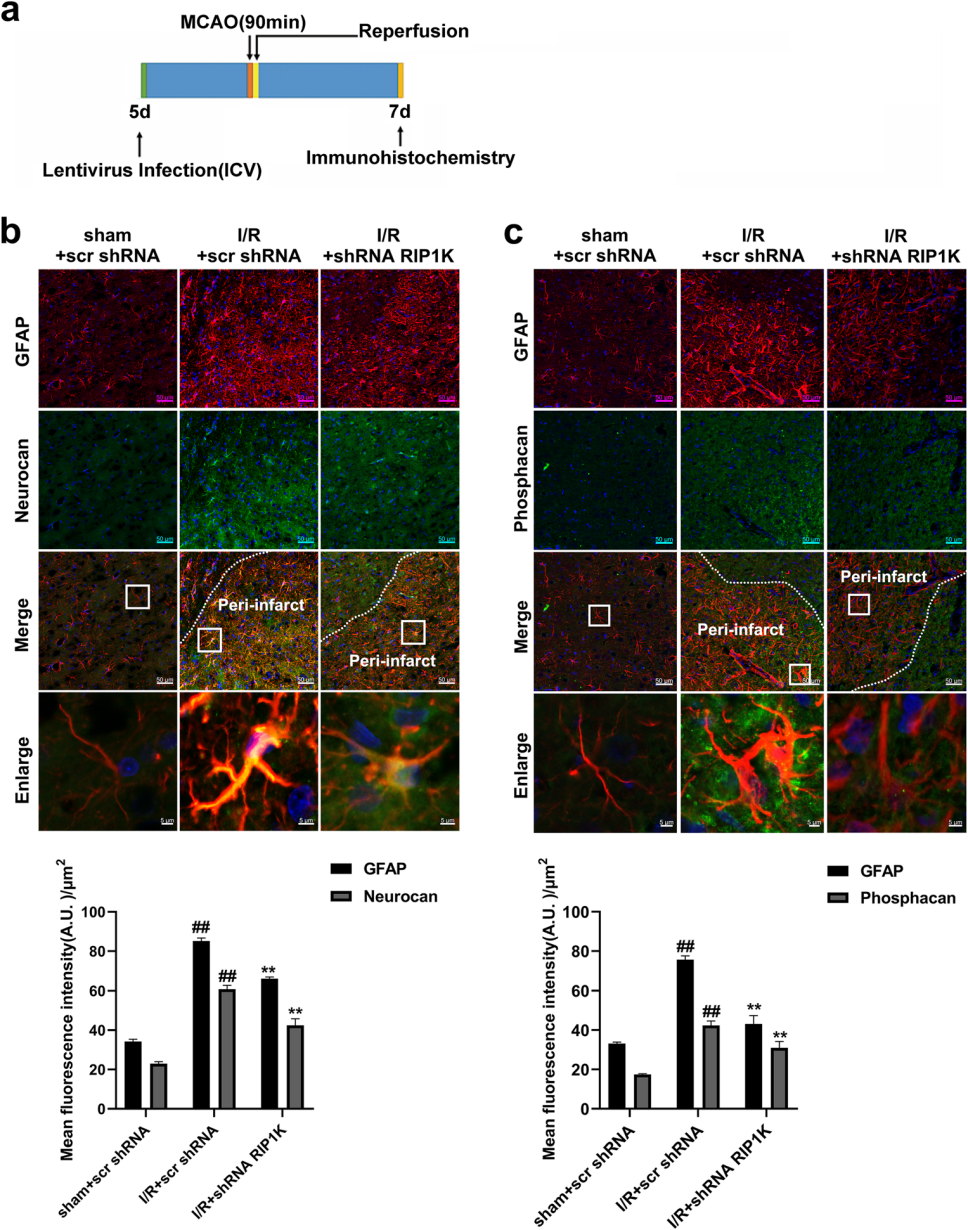
Knockdown of RIP1K reduces the protein level of neurocan and phosphacan in GFAP-positive cells after I/R. **a** Experimental protocol. **b** Representative images of double staining for GFAP (red) and neurocan (green) in the cerebral cortex of rats 7 days after I/R or in sham-operated rats. **c** Representative images of double staining for GFAP (red) and phosphacan (green) in the cerebral cortex of rats at day 7 after I/R or in sham-operated rats. Hoechst (blue) was used to stain the nucleus. The white dotted line represents the edge between the infarct area and the peri-infarct area, and the white boxes indicate the corresponding area of the enlarged images shown below. Data are mean ± SD, *n* = 3. ^##^*P* < 0.01 vs. sham + scr shRNA group; ^**^P < 0.01 vs. I/R + scr shRNA group. Statistical analysis was carried out with one-way ANOVA followed by a post hoc Tukey’s test

**Fig. 6:**
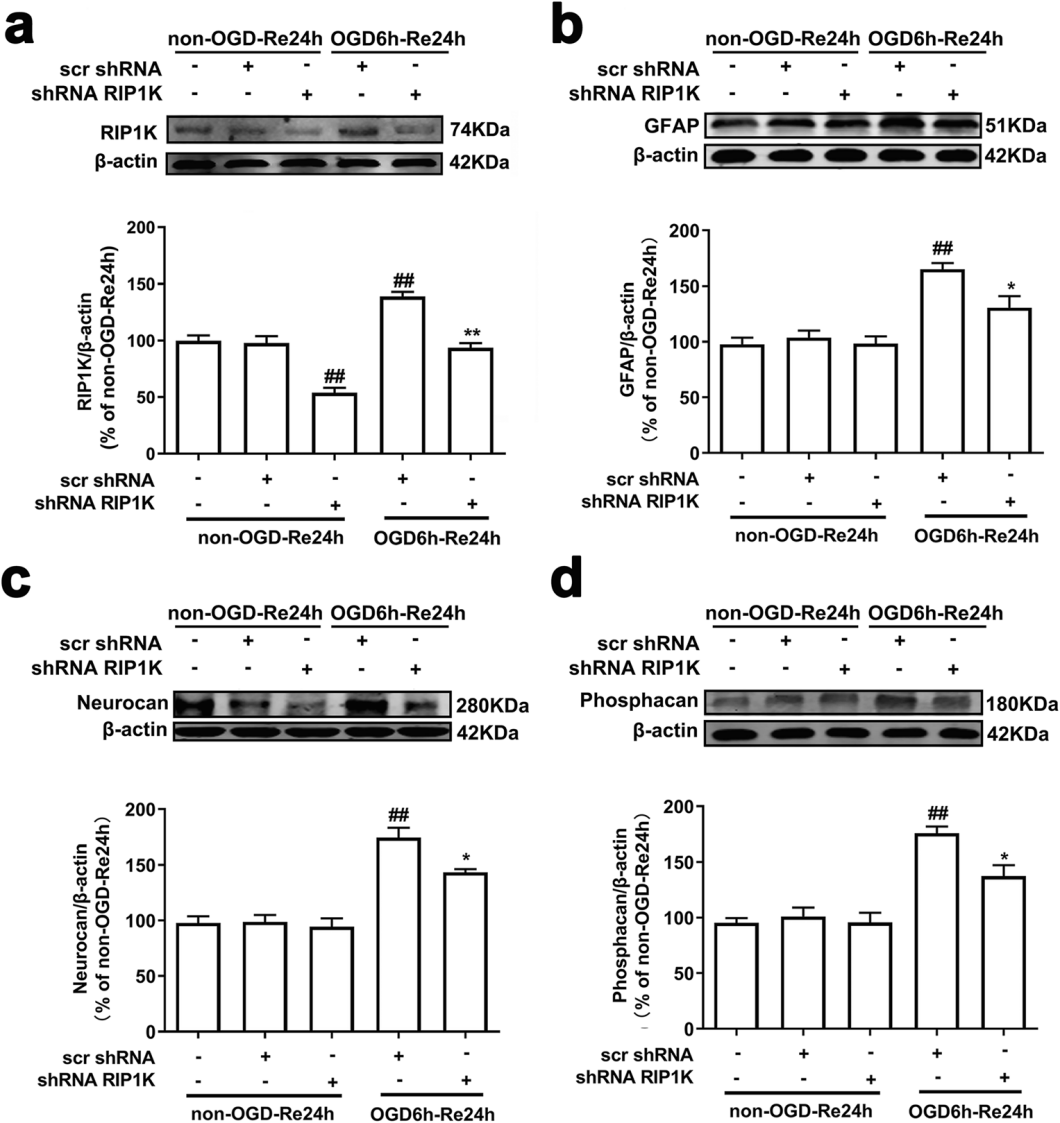
Knockdown of RIP1K reduces the protein level of RIP1K (**a**), GFAP (**b**), neurocan (**c**), and phosphacan (**d**) in astrocytes after OGD/Re injury with western blotting analysis. Astrocytes were exposed to OGD for 6 h followed by reoxygenation for 24 h. Data are mean ± SD, *n* = 3. ^##^*P* < 0.01 vs. non-OGD-Re24h + scr shRNA group; ^*^P < 0.05, ^**^P < 0.01 vs. OGD-6 h-Re24h + scr shRNA group. Statistical analysis was carried out with one-way ANOVA followed by a post hoc Tukey’s test

In most of CNS injuries, astrocytes have been reported giving a response to the tissue damage by undergoing rapid hypertrophy and hyperplasia [52]. The reactive astrocytes secrete some molecules, such as chondroitin sulfate proteoglycans (CSPGs) that not only inhibit the axonal regeneration at the site of the glial scar but also influence several neuronal and glial physiological properties through the modification of the extracellular microenvironment [53, 54]. However, the roles of RIP1K in the formation of astrogliosis and glial scar remain unknown. In the current study, we found that the knockdown of RIP1K markedly decreased the immunoreactivity of GFAP, as well as the immunostaining of neurocan (Fig. 5b) and phosphacan (Fig. 5c) in GFAP-positive cells in the peri-infarct region at day 7 after I/R injury. Consistent with the results in vivo, the knockdown of RIP1K could reduce the protein levels of RIP1K, GFAP, neurocan, and phosphacan in astrocytes at OGD6h/Re24h (Fig. 6).

### Delayed Administration of Nec-1 Suppresses Ischemic Stroke-Induced Formation of Astrogliosis and Glial Scar In Vivo and In Vitro

**Fig. 7:**
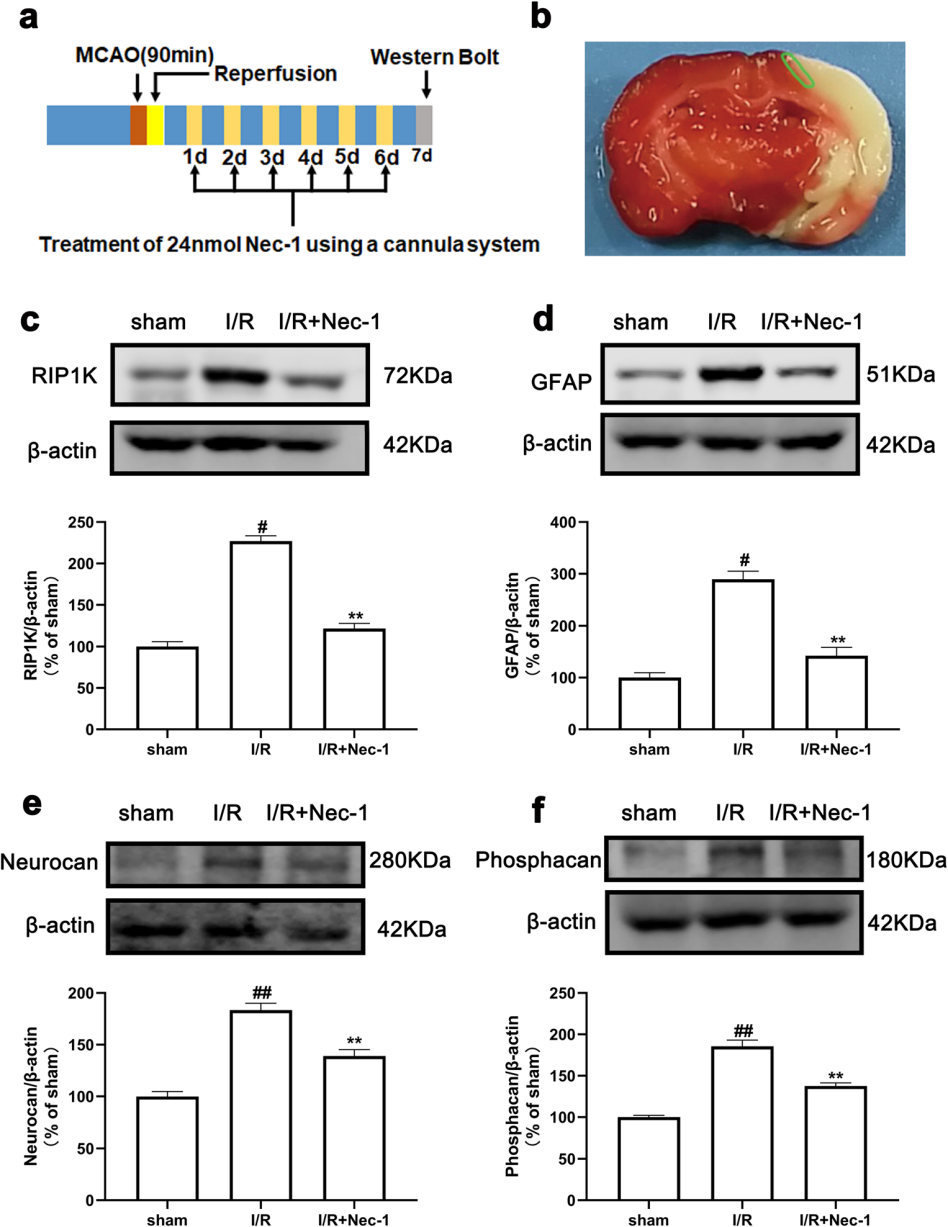
Delayed administration of Nec-1 reduces necroptosis-related protein and the glial scar marker after I/R with western blotting analysis. Transient ischemic stroke was induced by MCAO for 90 min followed by reperfusion (I/R) for 7 days. Nec-1 was injected (icv) for 7 days starting from 24 h post-reperfusion. **a** Experimental protocol. **b** Brain slice stained with 2,3,5-triphenyltetrazolium chloride (TTC). The region (the green line) indicates the peri-infarct regions of the ischemic cerebral cortex collected as samples for western blotting analysis. **c**–**f** Delayed administration of Nec-1 reduces the level of RIP1K and the glial scar markers GFAP, neurocan, and phosphacan. Data are mean ± SD, *n* = 3. ^#^*P* < 0.05, ^##^*P* < 0.01 vs. sham group; ^**^P < 0.01 vs. I/R group. Statistical analysis was carried out with one-way ANOVA followed by a post hoc Tukey’s test

**Fig. 8:**
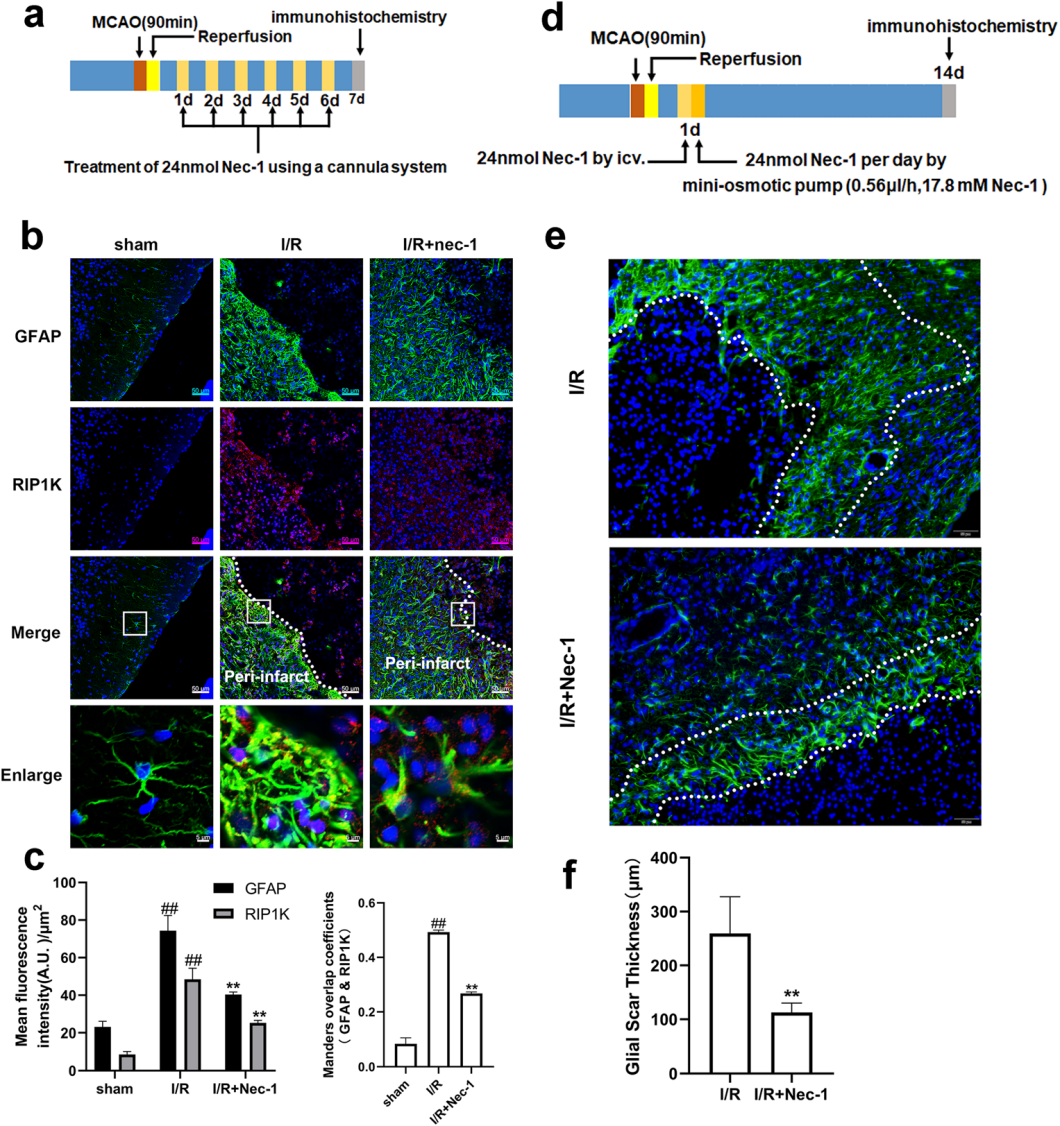
Delayed administration of Nec-1 reduces the formation of glial scar in the cerebral cortex of rats after I/R. Transient ischemic stroke was induced by MCAO for 90 min followed by reperfusion (I/R) for 7 or 14 days. Nec-1 was injected (icv) for 7 days (**a**, **b**, **c**) or for 14 days (**d**, **e**, **f**) starting from 24 h post-reperfusion. **a** Experimental protocol for **b**. **b** Representative images of double staining for GFAP (green) and RIP1K (red) in the cerebral cortex of rats 7 days after I/R or in sham-operated rats. Hoechst (blue) was used to stain the nucleus. The white dotted line represents the edge between the infarct area and the peri-infarct zones, and the white boxes indicate the corresponding area of the enlarged images shown below. Delayed administration of Nec-1 reduces the protein level of RIP1K in astrocytes. **c** Quantification of red fluorescence intensity of RIP1K and green fluorescence intensity of GFAP immunostaining. Mander’s overlap coefficient demonstrated the colocalization between GFAP and RIP1K in **b**. ^##^*P* < 0.01 vs. sham group; ^**^P < 0.01 vs. I/R group. Statistical analysis was carried out with one-way ANOVA followed by a post hoc Tukey’s test. **d** Experimental protocol for **e**. **e**, **f** Representative images of cerebral cortex double staining for GFAP (green) and RIP1K (red) in rats 14 days after I/R or in sham-operated rats. Hoechst (blue) was used to stain the nucleus. The white dotted line represents the glial scar. Delayed administration of Nec-1 reduces the thickness of glial scar induced by tMCAO. Data are mean ± SD, *n* = 6.^**^P < 0.01 vs. I/R group. Statistical analysis was carried out with Student’s *t* test

**Fig. 9:**
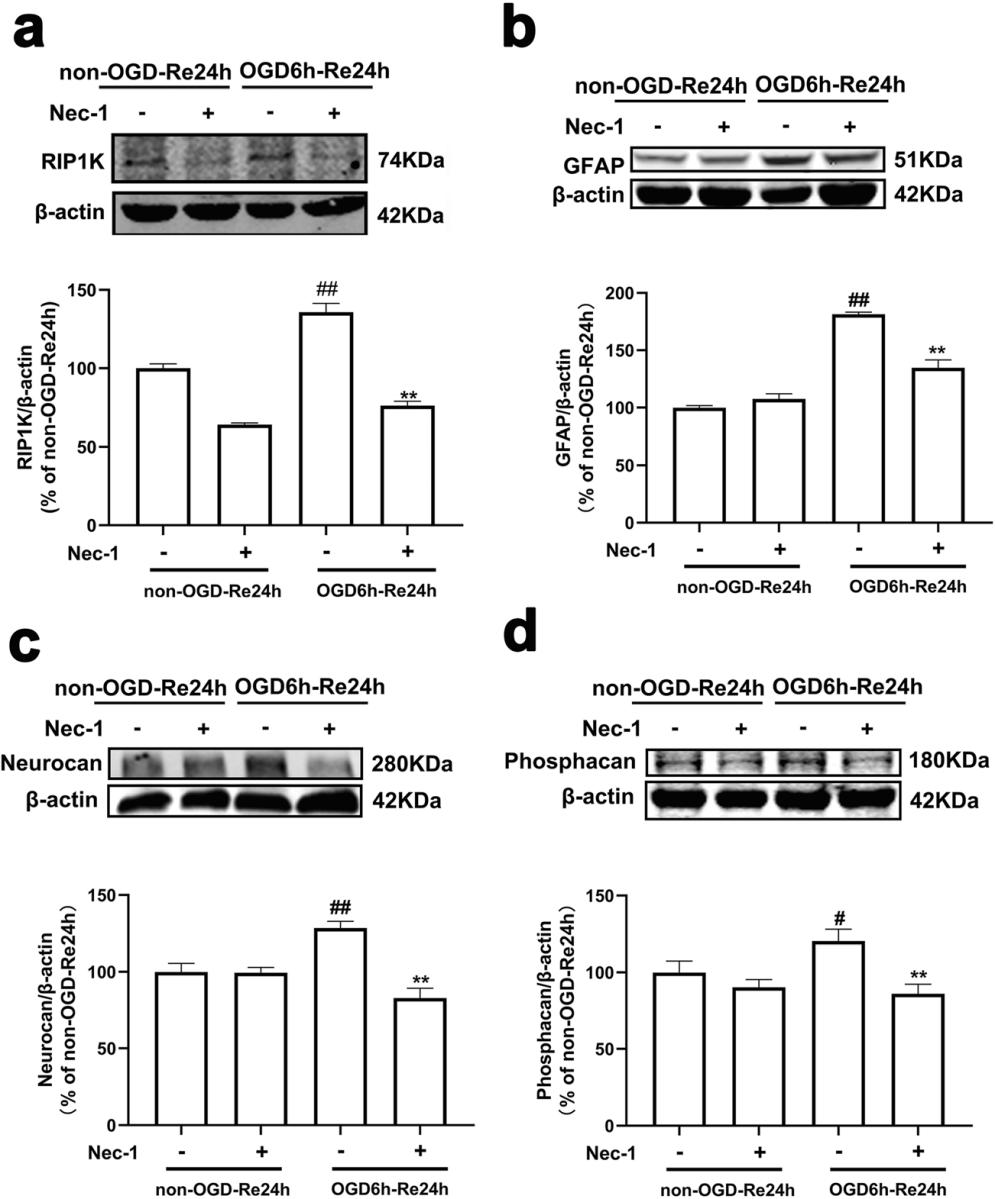
Delayed administration of Nec-1 upon reoxygenation reduces the protein level of RIP1K (**a**) and the glial scar markers GFAP (**b**), neurocan (**c**), and phosphacan (**d**) after OGD/Re injury. Astrocytes were exposed to OGD for 6 h followed by reoxygenation for 24 h. Nec-1 (100 μM) was added to the cells upon reoxygenation. Data are mean ± SD, *n* = 3. ^#^*P* < 0.05, ^##^*P* < 0.01 vs. non-OGD-Re24h group; ^**^P < 0.01 vs. OGD-6h-Re24h group. *β*-Actin protein was used as a loading control. Statistical analysis was carried out with one-way ANOVA followed by a post hoc Tukey’s test

We and others have previously demonstrated that Nec-1 or knockdown of RIP1K reduced the infarct size in the acute stage of ischemia in animals subjected to tMCAO or pMCAO injury [31, 55]. In order to exclude the possibility that the reduced GFAP, neurocan, and phosphacan expressions are not simply due to the reduced brain injury in the acute stage of ischemic stroke with RIPK inhibition, we next observed the effects of the delayed administration of Nec-1, a specific inhibitor of RIP1K, on the ischemic stroke-induced formation of astrogliosis and glial scar in vivo and in vitro. Firstly, we found that 24 nmol Nec-1, administered daily using the cannula system from day 1 (24 h) to day 7 after I/R, could dramatically decrease the RIP1K, GFAP, neurocan, and phosphacan levels (Fig. 7) and the colocalization of GFAP and RIP1K in the peri-infarct region at day 7 after I/R injury (Fig. 8a–c). Secondly, we used a mini-osmotic pump system to achieve continuous Nec-1 treatment for two weeks starting from 24 h after I/R and found that two weeks’ use of Nec-1 could observably attenuate the thickness of glial scar in the peri-infarct region at day 14 after I/R injury (Fig. 8d–f). Consistent with the results in vivo, Nec-1, added upon reoxygenation, also could attenuate the protein levels of RIP1K, GFAP, neurocan, and phosphacan in astrocytes exposed to OGD for 6 h and reoxygenation for 24 h (Fig. 9).

### Knockdown of RIP1K Suppresses OGD/Re-Induced Astrocyte Proliferation

**Fig. 10:**
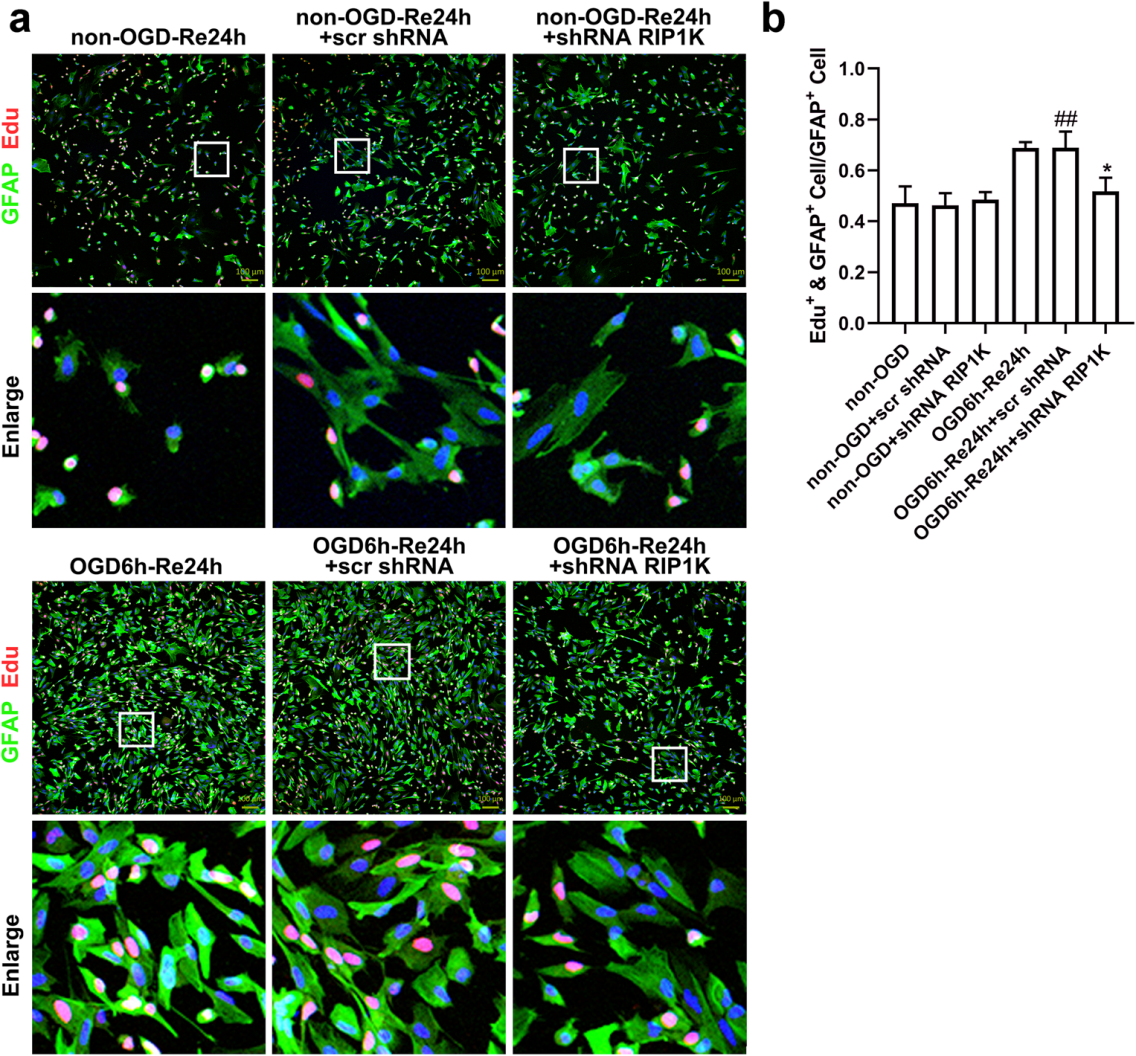
Knockdown of RIP1K attenuates OGD/Re-induced astrocyte proliferation. **a** Representative images of EdU (red) and GFAP (GFAP) double staining in astrocytes. The astrocytes were treated with OGD for 6 h and reoxygenation (OGD/Re) for 24 h. EdU (10 μm) was added to the cells after OGD and labeled with Apollo® 567 at 24 h after reoxygenation, and GFAP was used to label astrocytes. Hoechst (blue) was used to stain the nucleus. The white boxes indicate the corresponding area of the enlarged images shown below. **b** The percentage of proliferating astrocytes (EdU^+^&GFAP^+^ cells/GFAP^+^ cells) in **a**. Data are mean ± SD, *n* = 3. ^##^*P* < 0.01 vs. non-OGD-Re24h + scr shRNA group; ^*^P < 0.05 vs. OGD-6h-Re24h + scr shRNA group. Statistical analysis was carried out with one-way ANOVA followed by a post hoc Tukey’s test

To identify dividing cells, cultured astrocytes were added with the thymidine analog EdU using a saturating protocol to label all cells entering S phase [56]. After OGD 6 h/Re 24 h, nuclear EdU incorporation analysis showed the reduced number of proliferating cells in the astrocytes with knockdown of RIP1K (Fig. 10).

### Knockdown of RIP1K Suppresses Ischemia/Reperfusion or OGD/Re-Induced Reactive Astrogliosis and Glial Scar Formation via Down-Regulating VEGF-D/VEGFR-3 Signaling

**Fig. 11:**
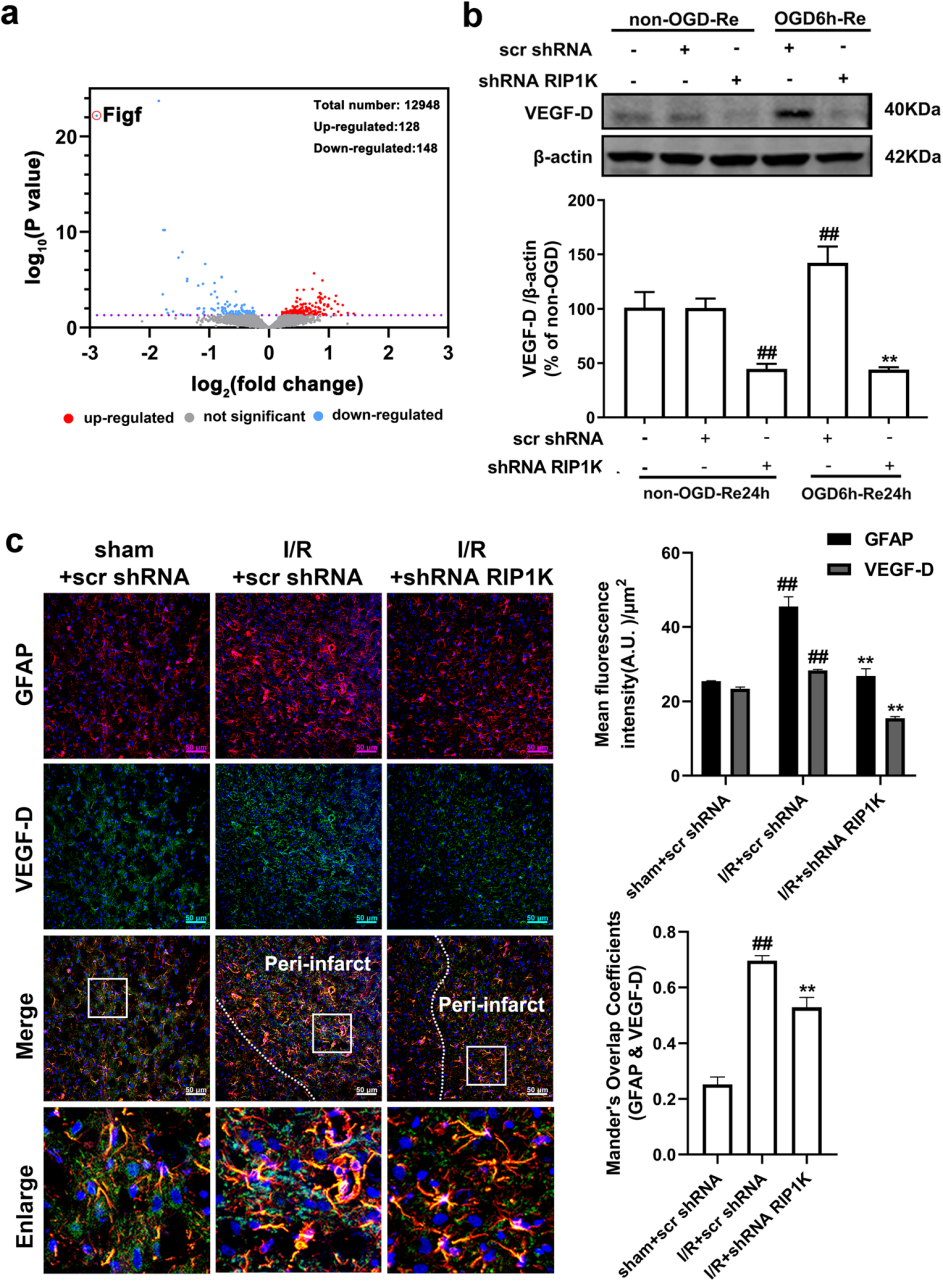
Knockdown of RIP1K reduces the gene and protein level of VEGF-D in astrocytes after I/R or OGD/Re. **a** Volcano plot of gene microarray analysis showed that 148 genes were down-regulated and 128 genes were up-regulated in RIPK1-knocked down astrocytes treated with OGD. *Figf*, the gene of VEGF-D, was found to be notably down-regulated (*n* = 3). **b** Knockdown of RIP1K reduces the protein level of VEGF-D in astrocytes after OGD for 6 h and reoxygenation for 24 h with western blotting analysis. Data are mean ± SD, *n* = 3. ^##^*P* < 0.01 vs. non-OGD-Re24h + scr shRNA group; ^**^P < 0.01 vs. OGD-6h-Re24h + scr shRNA group. **c** Representative images of cerebral cortex double staining for GFAP (red) and VEGF-D (green) in rats 7 days after I/R or in sham-operated rats. Hoechst (blue) was used to stain the nucleus. The white dotted line represents the edge between the infarct area and the peri-infarct area, and the white boxes indicate the corresponding area of the enlarged images shown below. Mander’s overlap coefficient demonstrated the colocalization between GFAP and VEGF-D. Data are mean ± SD, *n* = 3. ^##^*P* < 0.01 vs. sham + scr shRNA group; ^**^P < 0.01 vs. I/R + scr shRNA group. Statistical analysis was carried out with one-way ANOVA followed by a post hoc Tukey’s test

The VEGF family includes seven different homologous members, including VEGF-A, VEGF-B, VEGF-C, VEGF-D, VEGF-E, VEGF-F, and placental growth factor [41]. VEGF-D is secreted from the cell in a full-length form that can be proteolytically processed to a mature form with enhanced bioactivity. VEGF-D exerts its function through high affinity tyrosine kinase VEGFR-3. VEGFR-3 in the peri-infarction penumbra region is predominantly expressed in reactive astrocytes, suggesting that VEGFR-3 may be involved in the glial reaction during ischemic insults [41]. Further experiments found that, in a gene microarray of RIPK1 knockdown in OGD-treated astrocytes, 148 genes were down-regulated and 128 genes were up-regulated (Fig. 11a; Table S3). Unexpectedly, *Figf*, the gene of VEGF-D, was found to be notably down-regulated.

We next detected the effect of knockdown of RIP1K on the VEGF-D of reactive astrocytes in peri-infarction regions of cerebral cortex in rats at day 7 after I/R with immunohistochemistry analysis. In the astrocytes of sham-operated cerebral cortex, there were fine VEGF-D immunostaining. In astrocytes treated with I/R, the immunostaining of VEGF-D was significantly increased in GFAP-positive cells and knockdown of RIP1K could reverse I/R-induced increase in the immunoreactivity of VEGF-D in GFAP-positive cells (Fig. 11c). Similarly, the OGD/Re-induced up-regulation of VEGF-D in astrocytes was markedly blocked by knockdown of RIP1K (Fig. 11b). All these results revealed that knockdown of RIP1K can decrease the protein levels of VEGF-D in reactive astrocytes.

**Fig. 12:**
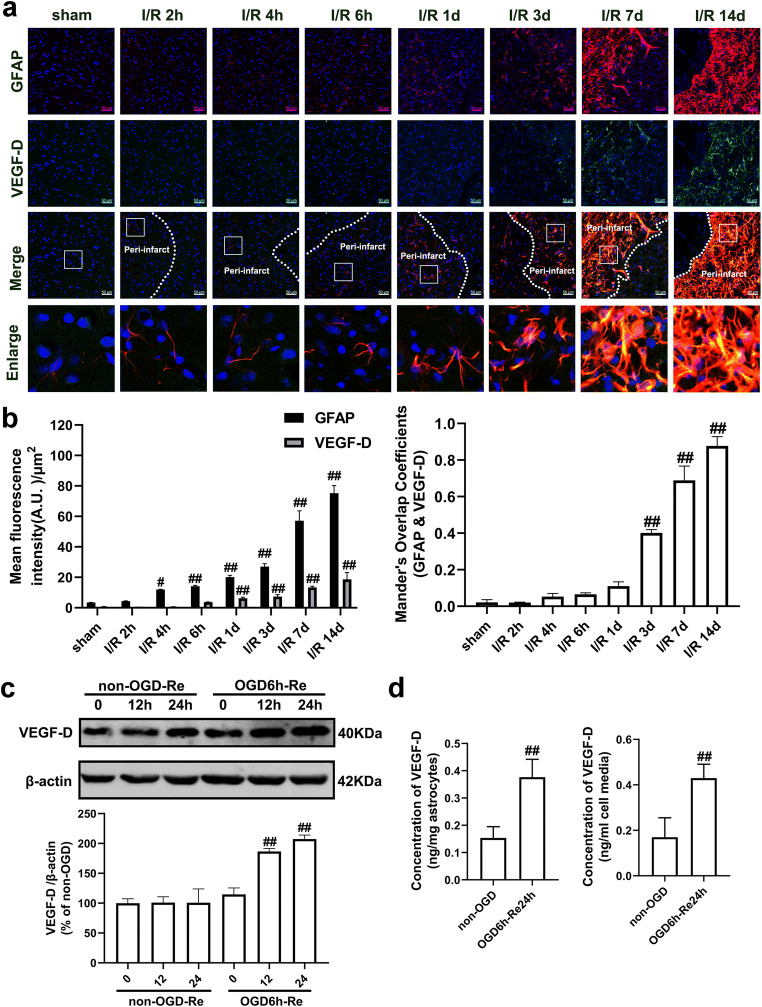
The VEGF-D level is increased in astrocytes after I/R injury or in astrocytes and cell cultured medium after OGD/Re injury. **a**, **b** The time-course changes of VEGF-D expression after I/R injury. Transient ischemic stroke was induced by MCAO for 90 min (I/R) followed by reperfusion (I/R). **a** Representative images of double staining for GFAP (red) and VEGF-D (green) in the cerebral cortex of rats. Hoechst (blue) was used to stain the nucleus. The white dotted line represents the edge between the infarct area and the peri-infarct area, and the white boxes indicate the corresponding area of the enlarged images shown below. **b** Quantification of green fluorescence intensity of VEGF-D and red fluorescence intensity of GFAP immunostaining in **a**. Mander’s overlap coefficient demonstrated the colocalization between GFAP and VEGF-D. Data are mean ± SD, *n* = 3. ^#^*P* < 0.05, ^##^*P* < 0.01 vs. sham group. **c** The time-course changes of VEGF-D level after OGD/Re injury with western blotting analysis. Astrocytes were exposed to OGD for 6 h followed by reoxygenation for 12 h or 24 h. Data are mean ± SD, *n* = 3. ^##^*P* < 0.01 vs. non-OGD group. **d** ELISA results showed that VEGF-D levels were increased both in astrocytes and in cell medium after OGD for 6 h and reoxygenation for 24 h. Data are mean ± SD, *n* = 3. ^##^*P* < 0.01 vs. non-OGD group. Statistical analysis was carried out with one-way ANOVA followed by a post hoc Tukey’s test (**b**, **c**) or with Student’s *t* test (**d**)

**Fig. 13:**
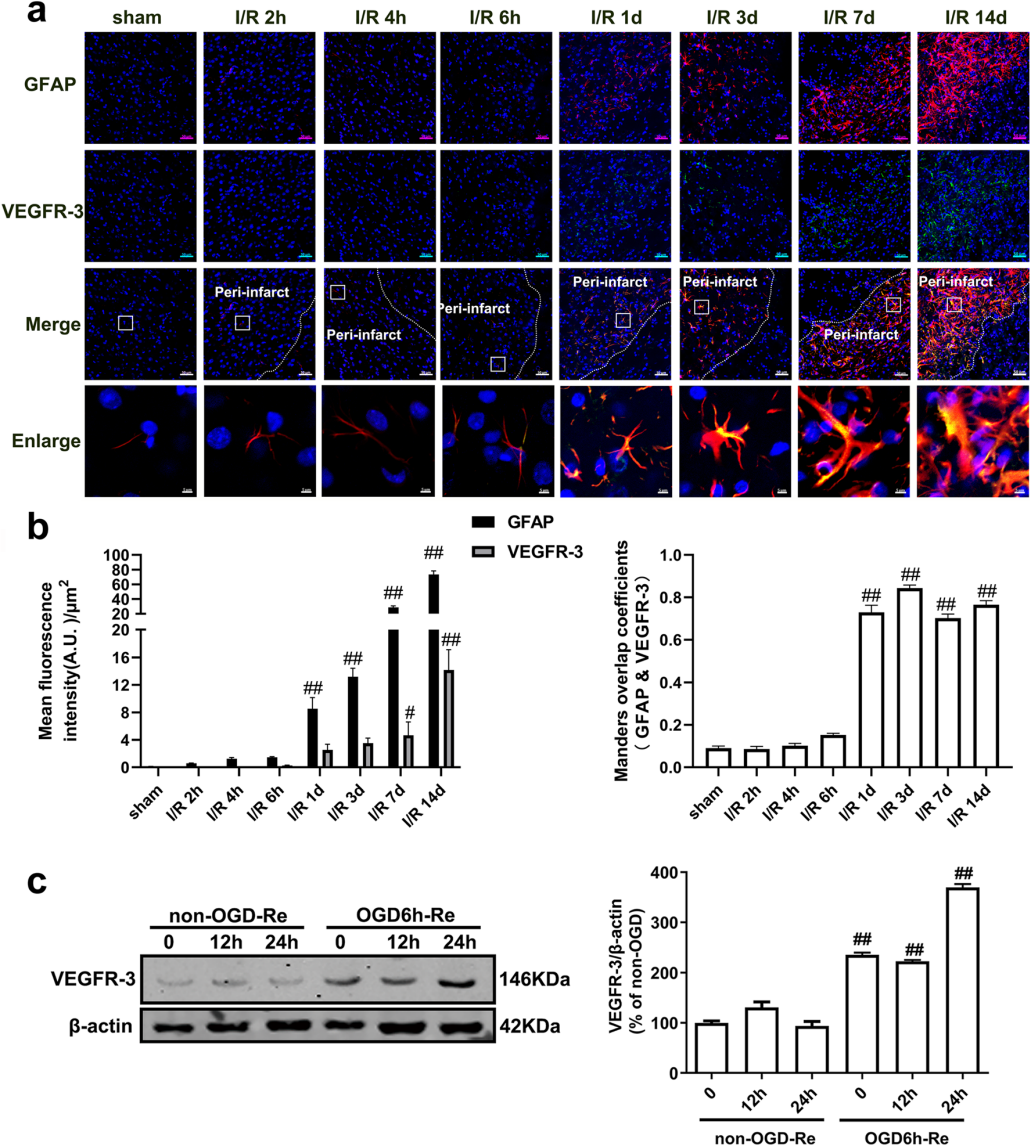
The VEGFR-3 level is increased in astrocytes after I/R injury or after OGD/Re injury. **a**, **b** The time-course changes of VEGFR-3 levels in astrocytes after I/R injury. Transient ischemic stroke was induced by MCAO for 90 min (I/R) followed by reperfusion (I/R) in rats. **a** Representative images of cerebral cortex double staining for GFAP (red) and VEGFR-3(green). Hoechst (blue) was used to stain the nucleus. The white dotted line represents the edge between the infarct area and the peri-infarct zones, and the white boxes indicate the corresponding area of the enlarged images shown below. **b** Quantification of green fluorescence intensity of VEGFR-3 and red fluorescence intensity of GFAP immunostaining in **a**. Mander’s overlap coefficient demonstrated the colocalization between GFAP and VEGFR-3. Data are mean ± SD, *n* = 3. ^#^*P* < 0.05, ^##^*P* < 0.01 vs. sham group. **c** The time-course changes of VEGFR-3 expression after OGD/Re injury with western blotting analysis. Astrocytes were exposed to OGD for 6 h followed by reoxygenation for 12 h or 24 h. Data are mean ± SD, *n* = 3. ^##^*P* < 0.01 vs. non-OGD group. Statistical analysis was carried out with one-way ANOVA followed by a post hoc Tukey’s test

**Fig. 14:**
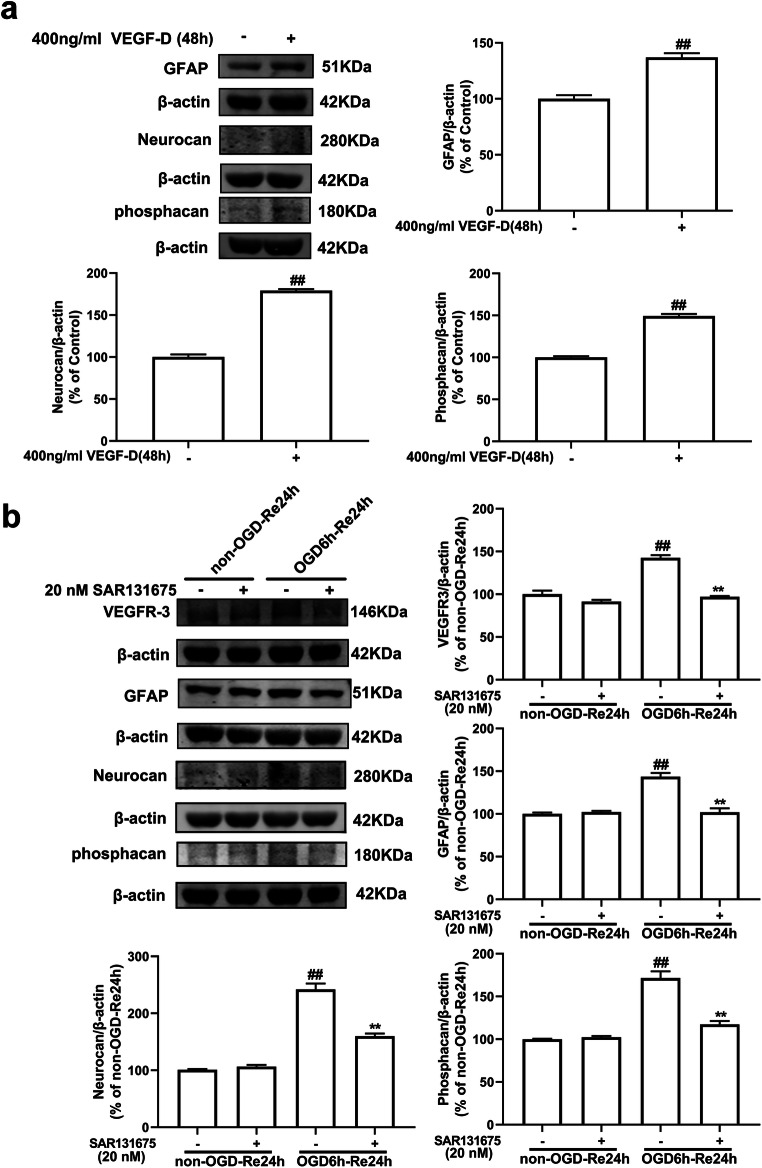
(a) Recombinant VEGF-D can induce the formation of glial scar. The astrocytes were treated with recombinant VEGF-D (400 ng/ml) for 48 h, and then, the levels of GFAP and phosphacan were detected with western blotting analysis. Data are mean ± SD, *n* = 3. ^##^*P* < 0.01 vs. non-OGD-Re24h group. Statistical analysis was carried out with Student’s *t* test. **b** SAR131675, a specific VEGFR-3 inhibitor reduces the levels of VEGFR-3, GFAP, neurocan, and phosphacan in astrocytes after OGD/Re with western blotting analysis. Astrocytes were exposed to OGD for 6 h followed by reoxygenation for 24 h. Astrocytes were treated with SAR131675 (20 nM) upon reoxygenation. Data are mean ± SD, *n* = 3. ^##^*P* < 0.01 vs. non-OGD group; ^**^P < 0.01 vs. OGD-6h-Re24h group. Statistical analysis was carried out with one-way ANOVA followed by a post hoc Tukey test

Therefore, we conducted a study on VEGF-D level in reactive astrocytes in the penumbra of cerebral cortex at 2 h, 4 h, 6 h, 1 d, 3 d, 7 d, and 14 d after reperfusion following MCAO for 90 min, based on GFAP and VEGF-D double immunohistochemistry staining. Our results revealed that no significant differences were found in VEGF-D level between the sham-operated group and the I/R 2 h group, I/R 4 h group, or I/R 6 h group. From 1 d to 14 d after focal ischemia, the expression of VEGF-D in GFAP-positive cells was significantly up-regulated over time (Fig. 12a, b). As shown in Fig. 13, the VEGFR-3 level in GFAP-positive cells was dramatically increased at 7 d and 14 d after I/R (Fig. 13a, b). We next observed the time-course changes of VEGF-D and VEGFR-3 in OGD/Re-induced astrocyte injury. Consistent with the results in vivo, OGD/Re induced an increase in VEGF-D (Fig. 12c) and VEGFR-3 (Fig. 13c) in astrocytes. ELISA results revealed that VEGF-D was increased both in cultured astrocytes and in the cell medium when astrocytes were exposed to OGD6h/Re24h, compared with the control group (Fig. 12d). Consistently, we also found that treatment with 400 ng/ml recombinant VEGF-D in astrocytes for 48 h could induce the formation of glial scar (Fig. 14a).

**Fig. 15:**
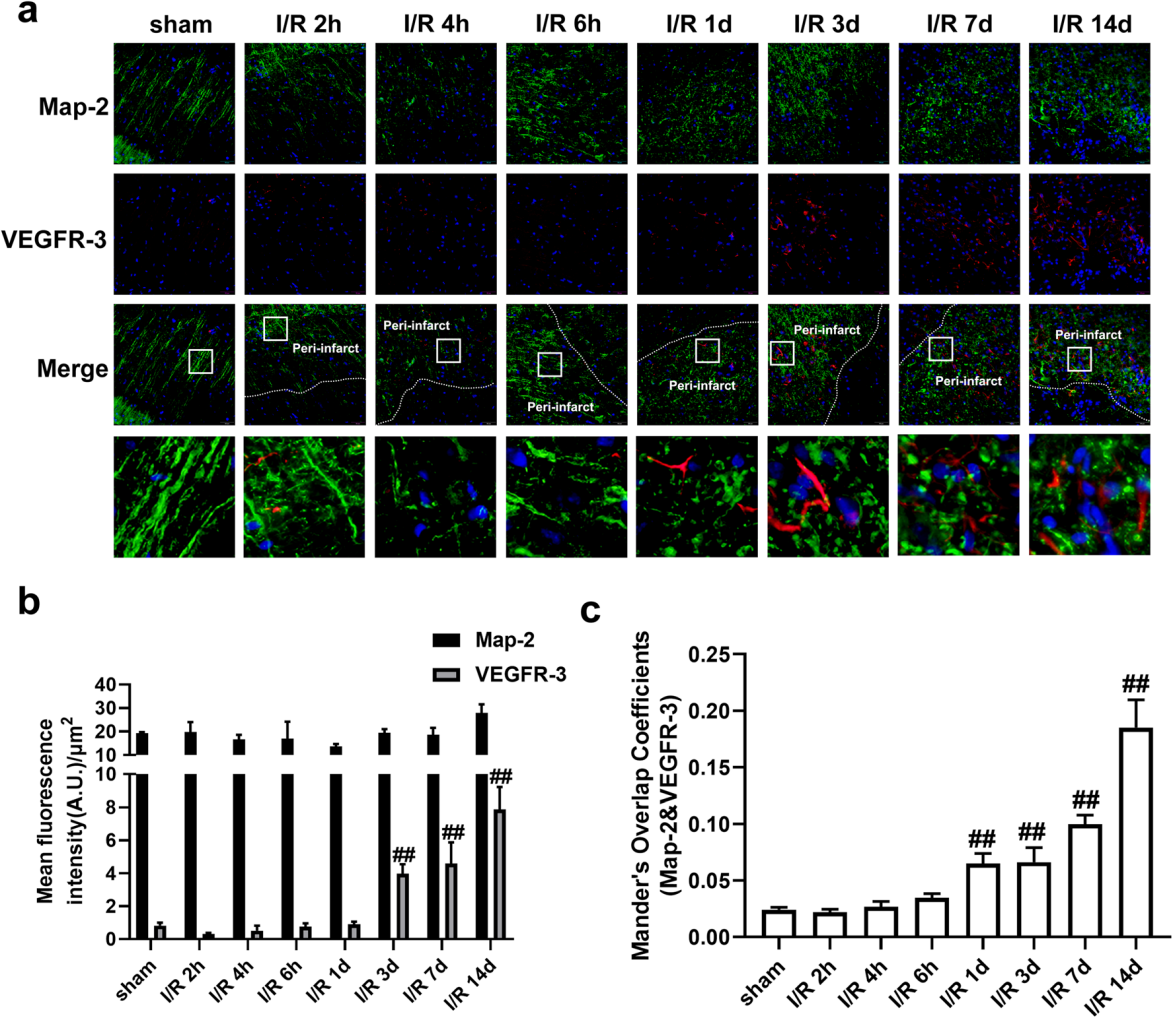
VEGFR-3 is almost absent in neurons. Transient ischemic stroke was induced by MCAO for 90 min (I/R) followed by reperfusion (I/R). **a** Representative images of double staining for Map-2 (green) and VEGFR-3 (red) in the cerebral cortex of rats at 2 h, 4 h, 6 h, 1 d, 3 d, 7 d, and 14 d after I/R or in sham-operated rats. Hoechst (blue) was used to stain the nucleus. The white boxes indicate the corresponding area of the enlarged images shown below. **b**, **c** Quantification of red fluorescence intensity of VEGFR-3 and green fluorescence intensity of Map-2 immunostaining. Mander’s overlap coefficient was applied to evaluate the colocalization between Map-2 and VEGFR-3 in **a**. When Mander’s overlap coefficient is less than 0.3, the correlation between the neuron (Map-2) and VEGFR-3 is considered to be low. Data are mean ± SD, *n* = 3. ^#^*P* < 0.05, ^##^*P* < 0.01 vs. sham group. Statistical analysis was carried out with one-way ANOVA followed by a post hoc Tukey’s test

SAR131675 is a potent and highly selective VEGFR-3 inhibitor; although it has moderate effects on VEGFR-2, it has 10 times greater selectivity for VEGFR-3 [57]. SAR131675 dependently inhibited the proliferation of primary human lymphatic cells, induced by the VEGFR-3 ligands VEGF-C and VEGF-D, with an IC_50_ of about 20 nM [57]. Next, we tested whether inhibition of VEGFR-3 by SAR131675 has an effect on the OGD/Re-induced reactive astrogliosis in vitro. In the OGD/Re group, the protein levels of GFAP and phosphacan were increased at OGD6h/Re24h. Surprisingly, SAR131675 could significantly reduce the protein levels of GFAP and phosphacan (Fig. 14b) at OGD6h/Re24h, suggesting that the VEGF-D/VEGFR-3 signaling may be involved in the glial reaction during ischemic insults. Interestingly, our results showed that there was scarcely VEGFR-3 expression in neurons after I/R with MAP-2 and VEGFR-3 double immunohistochemistry staining (Fig. 15).

All these results strongly suggested that VEGF-D/VEGFR-3 signaling is elevated in the reactive astrocytes and plays a crucial role in ischemic stroke-induced formation of astrogliosis and glial scar and knockdown of RIP1K can block the VEGF-D/VEGFR-3 signaling-induced astrogliosis and glial scar formation.

### Knockdown of RIP1K Protects OGD/Re-Induced Astrocytic Cell Death and Promotes the Neuronal Axonal Generation

**Fig. 16:**
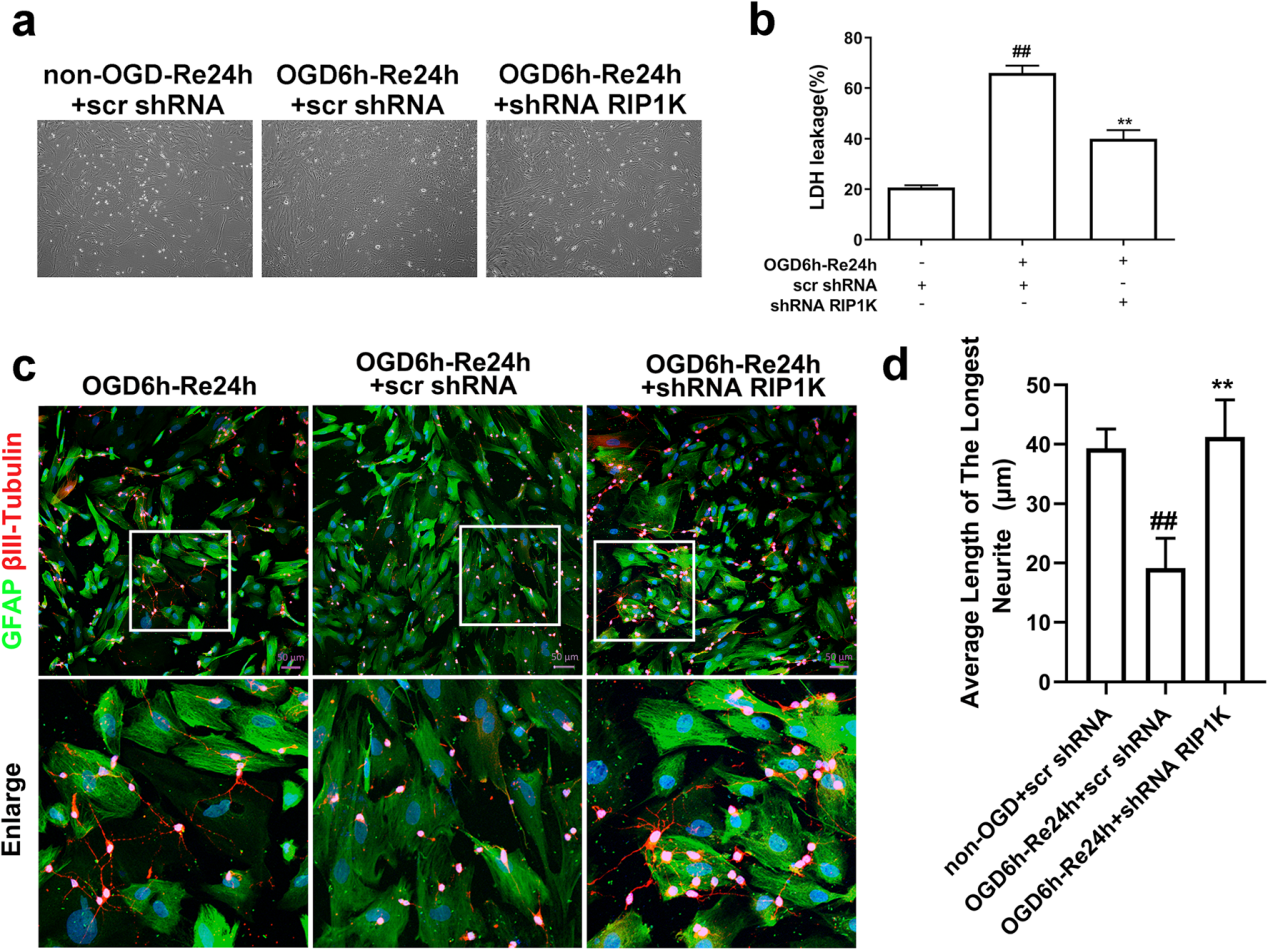
**a**, **b** Knockdown of RIP1K decreases LDH leakage of astrocytes. Astrocytes were exposed to OGD for 6 h followed by reoxygenation (OGD/Re) for 24 h. **a** Representative light microscope images of astrocytes. **b** shRNA RIP1K treatment decreases the LDH leakage of astrocytes. Data are mean ± SD, *n* = 3. ^##^*P* < 0.01 vs. non-OGD-Re24h + scr shRNA group; ^**^P < 0.01 vs. OGD-6h-Re24h + scr shRNA group. **c** Representative images of neurons (βIII-tubulin: red) and astrocytes (GFAP: green) were shown in a neuron and astrocyte co-culture system. Hoechst (blue) was used to stain the nucleus. Astrocytes were exposed to OGD for 6 h followed by reoxygenation (OGD/Re) for 24 h, and then, neurons were seeded and incubated together with astrocytes for additional 24 h. The white boxes indicate the corresponding area of the enlarged images shown below. **d** Quantification of average length of the longest neurite in **c**. Data are mean ± SD, *n* = 6. ^##^*P* < 0.01 vs. non-OGD-Re24h + scr shRNA group; ^**^P < 0.01 vs. OGD-6h-Re24h + scr shRNA group. Statistical analysis was carried out with one-way ANOVA followed by a post hoc Tukey’s test

Our prior studies demonstrated that RIP1K knockdown or Nec-1 treatment attenuated astrocytic necrotic cell death in the ischemic cerebral cortex [31]. In the present study, in an OGD6h/Re24h-induced glial scar formation model in primary cultured astrocytes, we also found that knockdown of RIP1K significantly protected the astrocytic cell death and reduced the LDH leakage of astrocytes (Fig. 16a, b). Second, as the greatest disadvantage of the formation of glial scar is blocking the regeneration of neurons [58, 59], we wondered if knockdown of RIP1K could promote the neurite growth and regeneration of neurons. Thus, we established the co-culture system of astrocytes and neurons, in which astrocytes were exposed to OGD for 6 h following reoxygenation for 24 h, and then, neurons were added and incubated together with astrocytes for additional 24 h. In this co-culture system, knockdown of RIP1K in astrocytes led to a great promoting effect on neurite growth of co-cultured neurons (Fig. 16c). The mean length of neurites showed a notable increase in the RIP1K knockdown group, compared with the non-knockdown RIP1K group (Fig. 16d). Therefore, our results revealed that knockdown of RIP1K in astrocytes facilitates the extension of neuronal axons co-cultured with astrocytes.

### Delayed Administration of Nec-1 Reduces Necrotic Cell in Lesion Area of Cerebral Cortex in tMCAO Rats

**Fig. 17:**
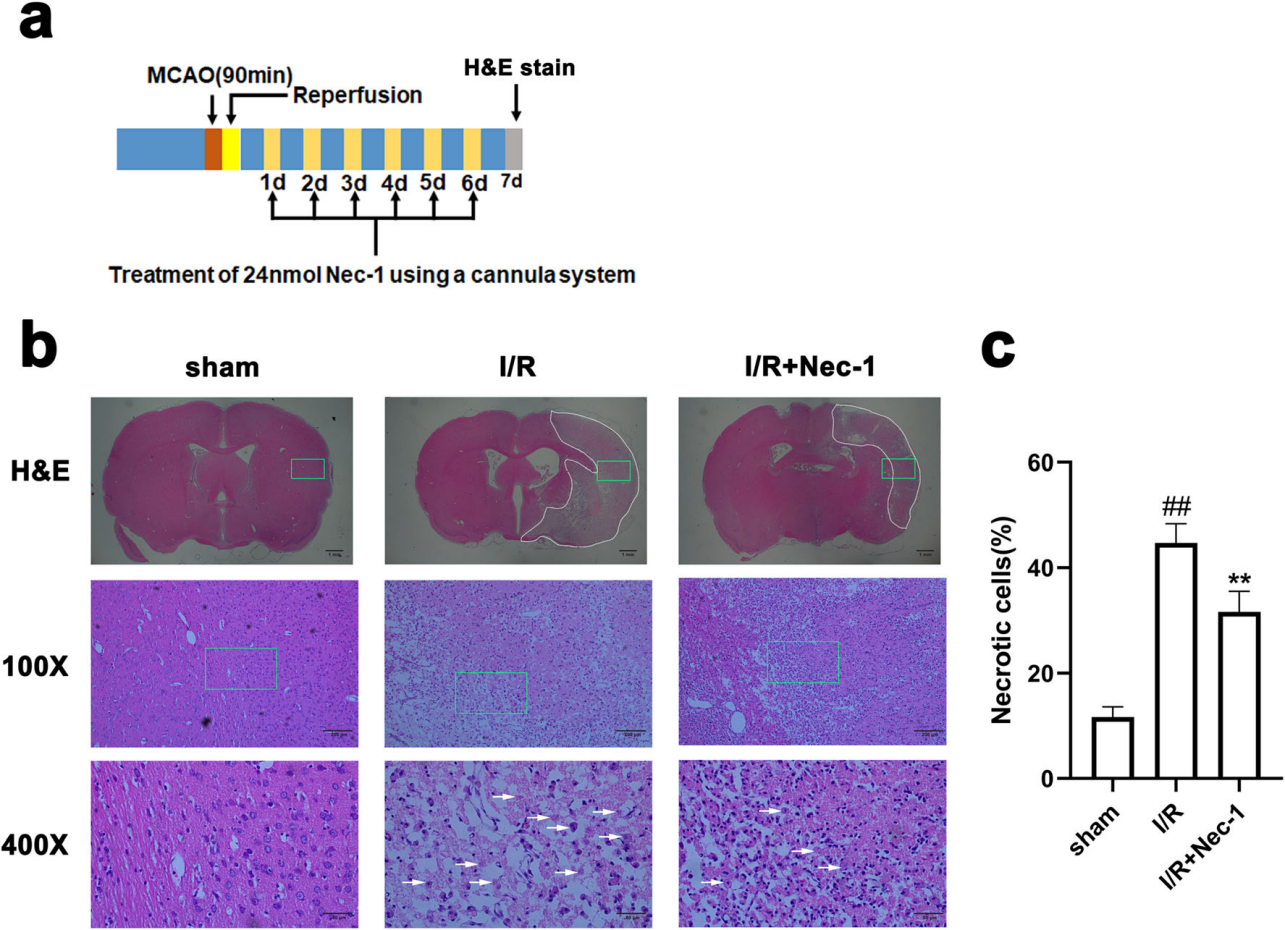
Delayed administration of Nec-1 reduces the necrotic-like cells in the cerebral cortex of rats after I/R. Transient ischemic stroke was induced by MCAO for 90 min followed by reperfusion (I/R) for 7 days. Nec-1 was injected (icv) for 7 days starting from 24 h post-reperfusion. **a** Experimental protocol. **b** Representative images of H&E staining in the cerebral cortex of rats at 7 days after I/R or in sham-operated rats. The area enclosed by a white dotted line is the lesion region, the green boxes indicate the corresponding area of the enlarged images shown below, and the white arrow points to necrotic-like cells. **c** Delayed administration of Nec-1 reduces the necrotic cells in the cerebral lesion region. Data are mean ± SD, *n* = 6. ^##^*P* < 0.01 vs. sham group, ^**^P < 0.01 vs. I/R group. Statistical analysis was carried out with one-way ANOVA followed by a post hoc Tukey’s test

When programmed necrosis occurs, cells are often accompanied by obvious morphological changes, such as swelling of cells, nuclear pyknosis, indiscernible nuclei, or loss of nuclei [46]. H&E staining showed delayed administration of Nec-1 for 7 days reduced the necrotic-like cells in the cerebral cortex of rats after I/R (Fig. 17).

### Knockdown of RIP1K Reduces Brain Atrophy and Ameliorates Behavioral Symptoms in the Recovery Phase of Rats after tMCAO

**Fig. 18:**
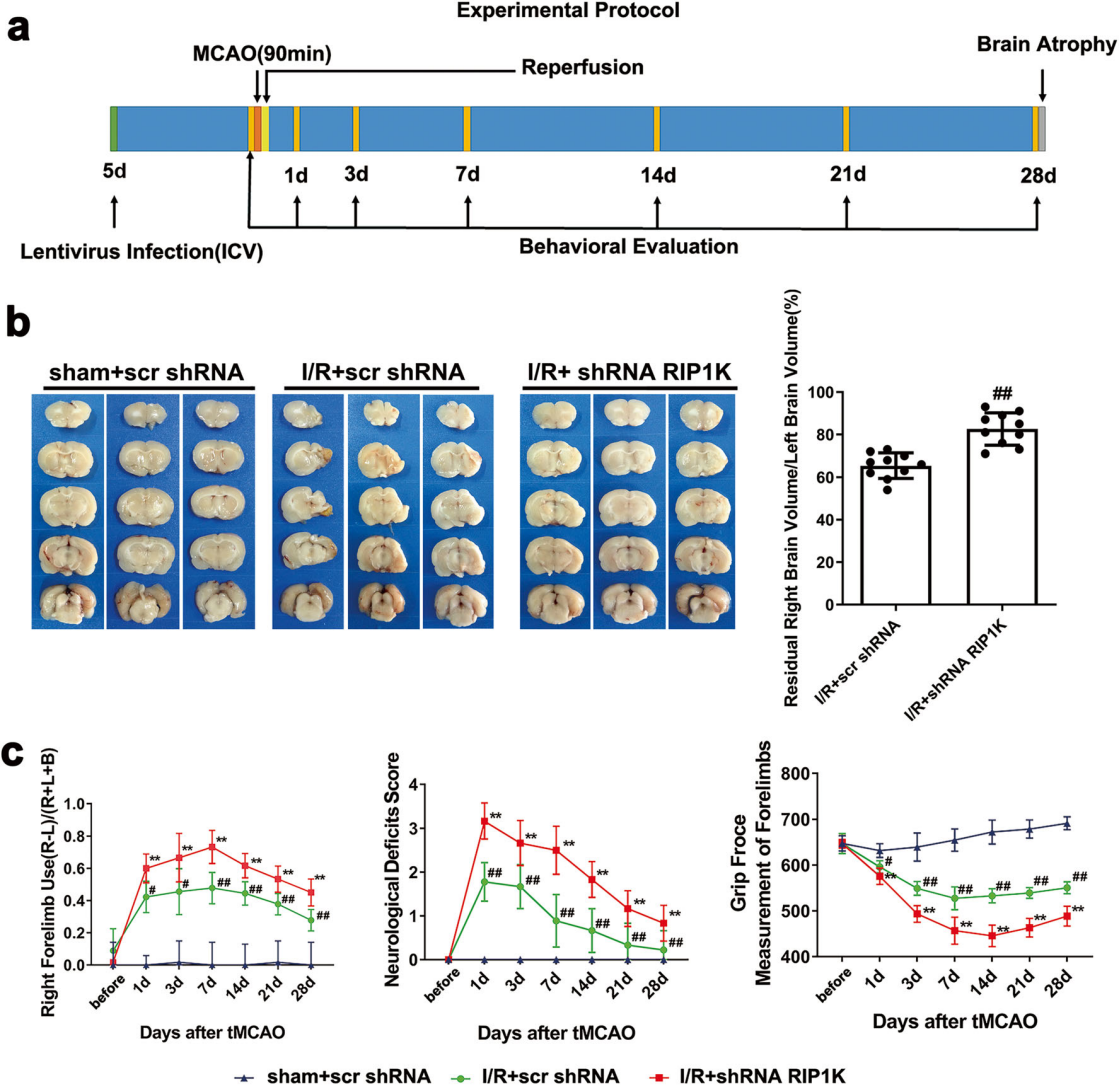
Knockdown of RIP1K promotes rats’ brain functional recovery after I/R injury. Transient ischemic stroke was induced by MCAO for 90 min (I/R) followed by reperfusion for 28 days (I/R). **a** Experimental protocol. Knockdown of RIP1K reduces brain atrophy (**b**) and improves the neurological deficits (**c**). Data are mean ± SD, *n* = 10. ^#^*P* < 0.05, ^##^*P* < 0.01 vs. sham + scr shRNA group. ^**^P < 0.01 vs. I/R + scr shRNA group. Statistical analysis was carried out with two-way ANOVA followed by a post hoc Tukey test for multiple comparisons and Student’s *t* test for two groups and multiple T tests which statistical significance determined using the Bonferroni-Dunn method for the neurological deficit evaluation

Recently, we reported that RIP1K knockdown or Nec-1 treatment reduced infarction volume and improved neurological deficits induced by pMCAO [31]. In the current study, we found that knockdown of RIP1K significantly attenuated the volumes of brain atrophy at 28 d after reperfusion following tMCAO for 90 min (Fig. 18b), decreased the right forelimb use, improved the neurological impairment score, and increased the grip strength from 1 d to 28 d following tMCAO (Fig. 18c). These results suggest that knockdown of RIP1K promotes the recovery of brain function in the late stage of brain injury after tMCAO.

### RIPK3 and MLKL Are Involved in Ischemia-Induced Reactive Astrogliosis

**Fig. 19:**
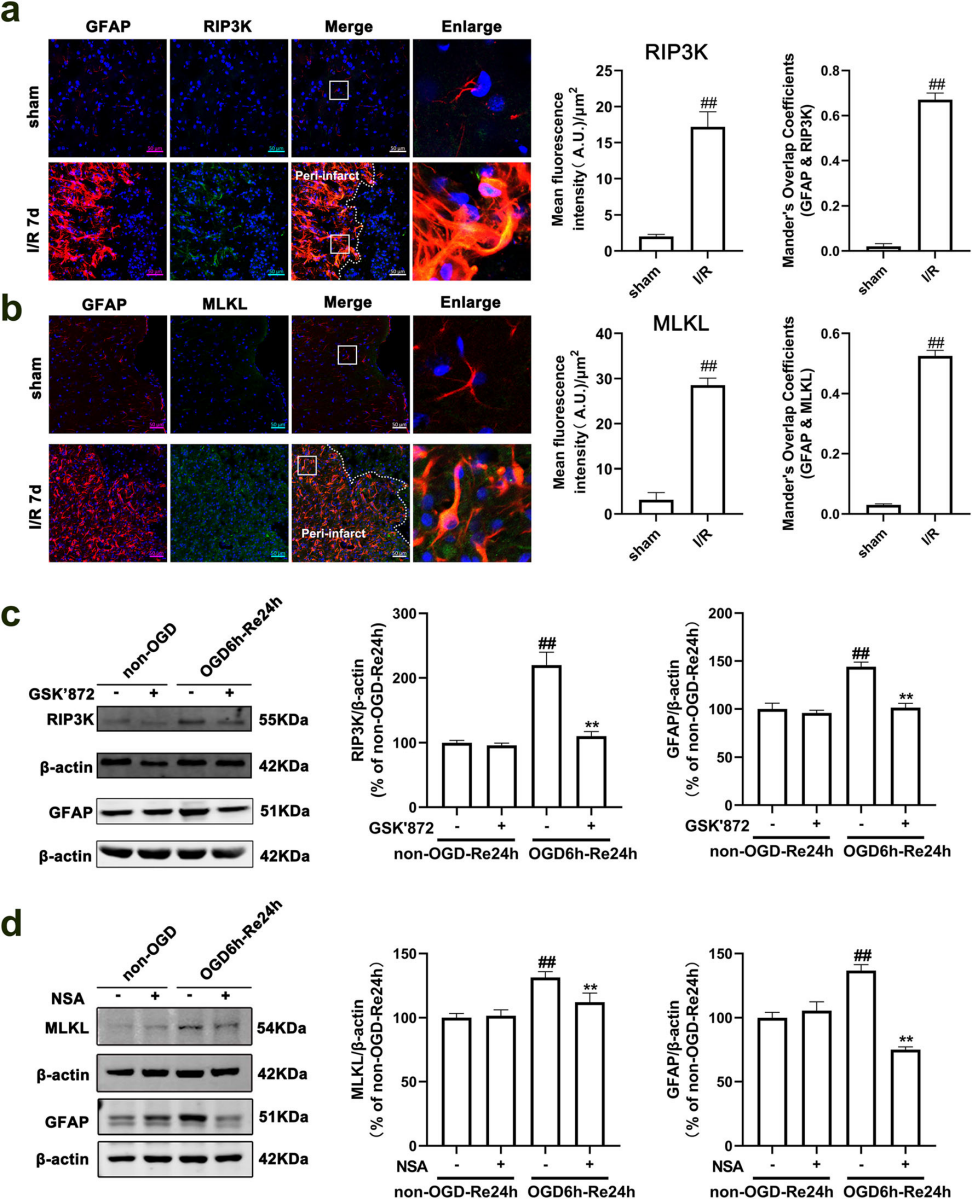
RIPK3 and MLKL are involved in ischemia-induced reactive astrogliosis. **a**, **b** Representative images of double staining for GFAP (red) and RIP3K or MLKL (green) in the cerebral cortex of rats 7 days after I/R or in sham-operated rats. Hoechst (blue) was used to stain the nucleus. The white dotted line represents the edge between the infarct area and the peri-infarct zone, and the white boxes indicate the corresponding area of the enlarged images shown below. Mander’s overlap coefficient demonstrated the colocalization between GFAP and RIP3K or MLKL. Data are mean ± SD, *n* = 3. ^##^*P* < 0.01 vs. sham group. Statistical analysis was carried out with Student’s *t* test. **c**, **d** Delayed administration of GSK′872 or NSA reduces the protein level of RIP3K or MLKL and GFAP after OGD/Re injury. Astrocytes were exposed to OGD for 6 h followed by reoxygenation for 24 h. GSK′872 (10 *μ*M) or NSA (1 *μ*M) was added to the cells upon reoxygenation. Data are mean ± SD, *n* = 3. ^##^*P* < 0.01 vs. non-OGD-Re24h group; ^**^P < 0.01 vs. OGD-6h-Re24h group. Statistical analysis was carried out with one-way ANOVA followed by a post hoc Tukey’s test

**Fig. 20:**
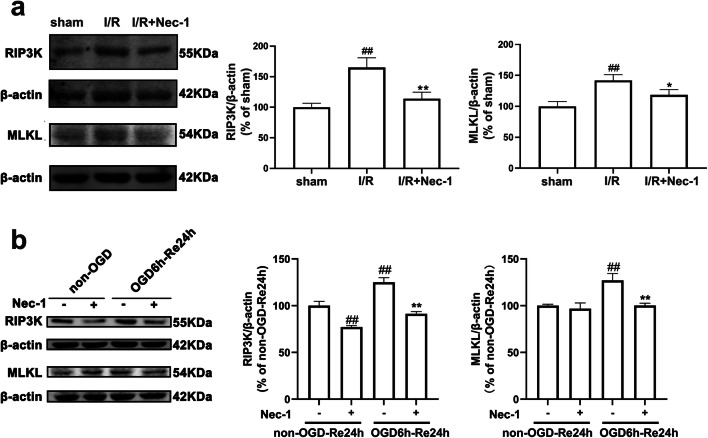
Delayed administration of Nec-1 reduces RIP1K downstream proteins RIP3K and MLKL following ischemia/reperfusion (I/R) or OGD/Re. **a** Delayed administration of Nec-1 reduces the protein level of RIP3K and MLKL after I/R. Transient ischemic stroke was induced by MCAO for 90 min followed by reperfusion (I/R) for 7 days. Nec-1 was injected (icv) for 7 days starting from 24 h post-reperfusion. **b** Delayed Nec-1 treatment reduces the protein level of RIP3K and MLKL after OGD/Re injury. Astrocytes were exposed to OGD for 6 h followed by reoxygenation for 24 h. Nec-1 (100 *μ*M) was added to the cells upon reoxygenation. Data are mean ± SD, *n* = 3. ^##^*P* < 0.01 vs. sham group; ^*^P < 0.01, ^**^P < 0.01 vs. I/R group. Statistical analysis was carried out with one-way ANOVA followed by a post hoc Tukey’s test

Necroptosis is a programmed process of necrosis, which is mediated together by RIP1K, RIP3K, and MLKL [60, 61]. We next tested whether RIP3K and MLKL, RIP1K downstream proteins, are involved in ischemia-induced reactive astrogliosis. The results of immunohistochemistry showed that RIP3K and MLKL were significantly increased in astrocytes in the peri-infarct region at day 7 after I/R injury (Fig. 19a, b). In addition, either RIP3K inhibitor GSK′872 or MLKL inhibitor NSA, added upon reoxygenation, reduced the protein level of GFAP (Fig. 19c, d). Further, delayed administration of Nec-1 decreased the protein levels of RIP3K and MLKL in the peri-infarct region at day 7 after I/R injury (Fig. 20a), and also Nec-1, added upon reoxygenation, could attenuate the protein levels of RIP3K and MLKL in astrocytes exposed to OGD for 6 h and reoxygenation for 24 h (Fig. 20b). These results revealed that RIPK3 and MLKL are also involved in ischemia-induced reactive astrogliosis.

## Discussion

Increasing studies have demonstrated that RIP1K is a crucial mediator of apoptotic and necrotic cell death as well as inflammatory pathways, resulting in that RIPK1 kinase has emerged as a promising therapeutic target for the treatment of a wide range of human diseases or conditions, including acute neuronal injury such as ischemic brain injury and traumatic brain injury and neurodegenerative, autoimmune, and inflammatory diseases. In the context of ischemic brain injury, Alexei Degterev and Junying Yuan firstly identified Nec-1 as a compound that blocks necrotic cell death in human and murine cells, and Nec-1 was shown to attenuate infarct volume in the transient middle cerebral artery occlusion (MCAO) model of stroke with a significant time window [33]. In a subsequent study, they further identified Nec-1 as an allosteric inhibitor of RIPK1 kinase activity [62]. More recently, we reported that RIP1K and RIP3K were up-regulated in the ischemic cortex following permanent MCAO and pharmacological or genetic inhibition of RIP1K decreased the infarction size [31]. We and others also found that genetic inhibition of RIP1K or Nec-1 reduced oxygen/glucose deprivation-induced death in primary neurons or astrocytes [31]. However, whether RIP1K is involved in the formation of astrogliosis and glial scar in CNS diseases including ischemic stroke is still largely unclear. Our current study provides the first evidences that (1) RIP1K activation is required for astrogliosis and glial scar formation after ischemic stroke; (2) the VEGF-D/VEGFR-3 signaling is elevated in the reactive astrocytes and plays a crucial role in ischemic stroke-induced formation of astrogliosis and glial scar; (3) RIP1K enhances the VEGF-D/VEGFR-3 signaling pathways contributing to astrogliosis and glial scar formation; (4) RIPK3 and MLKL are also involved in ischemia-induced reactive astrogliosis; and (5) inhibition of RIP1K promotes the brain functional recovery at least partially through inhibiting the formation of astrogliosis and glial scar via suppressing the VEGF-D/VEGFR-3 signaling pathways.

The hallmarks of reactive astrogliosis after ischemic injury include a pronounced increase in expression of GFAP and astrocyte hypertrophy. In the present study, we confirmed that GFAP was significantly increased at 7 days of I/R and a dense glial scar was formed at 14 days of I/R. Neurocan and phosphacan, the other markers of glial scar, were also markedly increased at 7 days of I/R, which is consistent with our previous research [45]. One novel finding of the current study is that RIP1K, a crucial mediator of necroptosis [33, 62], participates in the formation of astrogliosis and glial scar after ischemic stroke. RIP1K was significantly elevated in the reactive astrocytes in the peri-infarct cerebral cortex following tMCAO, and the time-point changes of RIP1K up-regulation were positively correlated to those of the formation of astrogliosis and glial scar. Thus, what is the role of elevated RIP1K during the formation of astrogliosis and glial scar? Further experiments revealed that knockdown of RIP1K significantly decreased the immunoreactivity of GFAP, neurocan, and phosphacan in the reactive astrocytes in the peri-infarct region at 7 days of subacute stage after I/R injury and decreased OGD/Re-induced increase in protein levels of GFAP, neurocan, and phosphacan in the reactive astrocytes in vitro. Proliferation and hypertrophy of astrocyte in the penumbra of ischemia are prominent features of ischemic stroke [6]. After OGD 6 h/Re 24 h, nuclear EdU incorporation results showed a reduced number of proliferating astrocytes with knockdown of RIP1K. These data provide strong evidence that RIP1K is crucially involved in regulating ischemic stroke-induced reactive astrocyte dynamics to control astrogliosis and glial scar, and pharmacologic or genetic inhibition of RIP1K in astrocytes is associated with reduced reactive astrogliosis.

We and others have previously demonstrated that Nec-1 or knockdown of RIP1K reduced the infarct size in the acute stage of ischemia in animals subjected to tMCAO or pMCAO injury [31, 55]. Our current studies further clarified that the reduced GFAP, neurocan, and phosphacan expressions are not simply due to the reduced brain injury in the acute stage of ischemic stroke with RIPK inhibition. This was evidenced by the fact that delayed administration of Nec-1 starting from 24 h post-ischemia for consecutive 7 days or 14 d could decrease the GFAP level and attenuate the thickness of glial scar in the peri-infarct region, respectively, indicating that RIPK inhibition plays a crucial role in reducing the astrogliosis and glial scar formation, at least, partially independent of its neuroprotective effect in the acute stage of ischemic injury.

What is the mechanism for Nec-1 or knockdown of RIP1K in reducing the reactive astrogliosis? We previously found that inhibition of RIP1K by Nec-1 or by shRNA RIP1K could directly protect neurons or astrocytes against OGD-induced cell injury [31]. Therefore, we speculated that one of the reasons for the reduced reactive astrogliosis mediated by knockdown of RIP1K or by Nec-1 might result from reducing astrocyte death and preserving normal astrocyte function in ischemic brains. Expectedly, knockdown of RIP1K directly protected OGD/Re-induced astrocytic cell death. Next, we unexpectedly found that the reduced reactive astrogliosis by knockdown of RIP1K or by Nec-1 was associated with repressing the VEGF-D/VEGFR-3 signaling pathways in astrocytes. In the brain, VEGF mediates angiogenesis, neural migration, and neuroprotection. However, excessive VEGF disrupts intracellular barriers, increases leakage of the choroid plexus endothelia, evokes edema, and activates the inflammatory pathway [40]. VEGF-D exerts its function through high affinity tyrosine kinase VEGFR-3. VEGFR-3 in the peri-infarction penumbra region is predominantly expressed in reactive astrocytes, suggesting that VEGFR-3 may be involved in the glial reaction during ischemic insults [41]. In the current study, we found that in a gene microarray of RIPK1 knockdown in OGD-treated astrocytes, 148 genes were down-regulated and 128 genes were up-regulated. Unexpectedly, *Figf*, the gene of VEGF-D, was found to be the most down-regulated gene. Further studies revealed that VEGF-D and VEGFR-3 were up-regulated in reactive astrocytes in the peri-infarct region and also in the OGD/Re-induced reactive astrogliosis in vitro. Importantly, VEGF-D was increased in the medium of cultured astrocytes, and exogenous recombinant VEGF-D could induce glial scar formation. Further studies demonstrated that SAR131675, a highly selective inhibitor of VEGFR-3, suppressed OGD/Re-induced reactive astrogliosis in vitro, which targets VEGFR-3 when the concentration is 20 nM. What is more, VEGFR-3 is almost absent in neurons and is highly expressed in astrocytes. These data indicate that VEGF-D/VEGFR-3 signaling in astrocyte might contribute to reactive astrocytes during ischemic stroke insults. We think this might result from paracrine or autocrine effects of VEGF-D in astrocytic responses to ischemic injury, leading to activation of VEGFR-3 signaling of astrocytes. In contrast, knockdown of RIP1K could reverse tMCAO-induced increase in the immunoreactivity of VEGF-D in reactive astrocytes of the peri-infarct region in vivo and decrease OGD/Re-induced increase in the protein levels of VEGF-D in reactive astrocytes in vitro. These data indicate that the reduced reactive astrogliosis mediated by knockdown of RIP1K or by delayed Nec-1 treatment is at least partially associated with protecting astrocytic cell injury and inhibiting the VEGF-D/VEGFR-3 signaling pathways of astrocytes.

Recent findings showed that attenuating reactive astrogliosis blocked brain injury after acute ischemic stroke [27, 30]. Consistent with this point of view, we further found that knockdown of RIP1K or Nec-1 delayed treatment could promote the neuronal axonal generation in glial scar in a neuron and astrocyte co-culture system in vitro and reduced the necrotic cells in lesion region of cerebral cortex and the volume of brain atrophy at late stage of 28 days after I/R injury. These data suggested that RIP1K activity is an important contributing factor for astrocyte dysfunction and reactive astrogliosis, and targeting RIP1K presents a better strategy in maintaining normal astrocytic function and suppressing the VEGF-D/VEGFR-3 signaling pathways in astrocytes, leading to blocking the excessive reactive astrogliosis and improving brain function after ischemic stroke. However, we cannot exclude the possibility that this may also attribute to reduced brain injury in the acute stage of ischemic stroke with RIPK inhibition. In addition, the present study also revealed that RIP1K downstream proteins RIP3K and MLKL may play an important role in the ischemic stroke formation of glial scars. Whether RIPK induces formation of glial scars in a RIP3K- and MLKL-dependent manner remains to be investigated.

Contrary to prevailing point of views and our present findings, more recently, Anderson et al. reported that astrocyte scar formation aids rather than prevents CNS axon regeneration in severe spinal cord injury (SCI) [63]. In their study, to be devoid of astrocytes, they used two loss-of-function transgenic mouse models that either selectively kills proliferating scar-forming astrocytes or deletes STAT3 signaling selectively from astrocytes which plays a crucial role in astrocyte scar formation after adult murine SCI. In contrast, in our current study, we indirectly inhibited the formation of astrocyte scar and did not delete astrocytes. Therefore, we think the contrary results between Anderson et al.’s and ours are mainly due to the degree of inhibition of the astrocyte scar, and in our point of view, moderate inhibition of astrocyte scar after CNS injury may aid CNS axon regeneration, but over-inhibition of astrocyte scar may prevent CNS axon regeneration.

Nec-1 is now widely used to block RIPK1 kinase activity in various experimental disease models, including stroke [33, 62]. However, in 2013, Alexei Degterev and Junying Yuan further found that necrostatin has effects other than blocking RIPK, e.g., Nec-1 also inhibits indoleamine-2,3-dioxygenase (IDO) [64]. The IDO-kynurenine pathway is activated under inflammatory conditions and leads to modulation of the innate and adaptive immune system, but is also implicated in neuroprotection [62]. Therefore, in the current study, we cannot rule out the involvement of IDO inhibition by Nec-1 in Nec-1-mediated inhibitory effects of astrocyte scar.

The protective effects of Nec-1 on the other cell types with activated RIP1K, such as microglia, endothelial cells, and neurons, have been reported by others. Huang et al. found microglia experienced RIP1- and RIP3-dependent necroptosis in the retinal degenerative rd1 mice and in the acute retinal neural injury mice, leading to microglia necroptosis-mediated inflammation. Necroptosis blockade using necrostatin-1 could suppress microglia-mediated inflammation, rescue retinal degeneration, or prevent neural injury [65]. Naito et al. reported that after MCAO induction, RIP1K was increased in CD31-positive endothelial cells and in NeuN-positive neurons, and inhibition of RIPK1 kinase can block the death of endothelial cells and neurons, as well as the production of proinflammatory cytokines and chemokines, while blocking necroptosis may only partially rescue cell death and amplification of the inflammatory response after the onset of reperfusion [66]. Our present discovery together with other’s findings indicates that activated RIP1K contributes to the necroptosis of all kinds of brain cells, including neurons, astrocytes, microglia, and endothelial cells upon brain injury, and necroptosis blockade therefore emerged as a novel therapeutic strategy for ameliorating neural injury such as ischemic stroke.

## Conclusions

In summary, we show for the first time that astrocytic RIP1K plays a critical role in reactive astrogliosis and ischemic astrocytic damage after ischemic stroke in vivo and in vitro. By using knockdown of RIP1K in rats’ brain and in primary astrocytes or Nec-1, a specific inhibitor of RIP1K, we have demonstrated that knockdown of RIP1K or delayed Nec-1 application reduces necrotic cells and brain atrophy, ischemic astrocytic damage, and reactive astrocyte formation, promotes the neuronal axonal generation in glial scar, and improves brain function; VEGF-D/VEGFR-3 signaling in astrocyte might be involved in the reactive astrocytes during ischemic stroke insults; and knockdown of RIP1K blocks VEGF-D/VEGFR-3 signaling in astrocyte associated with its inhibition of reactive astrogliosis. RIP1K downstream proteins RIP3K and MLKL may also play an important role in the ischemic stroke formation of glial scars. Additional studies are warranted for a more complete understanding of the underlying mechanisms regarding whether RIP1K regulates VEGF-D/VEGFR-3 signaling via RIP3K- and MLKL-dependent manner in astrocytes.

## Supplementary Information

ESM 1 (DOC 109 kb)

## Data Availability

The data generated for this study will be available upon reasonable request.
