# Supplementary material for: The Key Regulator of Necroptosis, RIP1 Kinase, Contributes to the Formation of Astrogliosis and Glial Scar in Ischemic Stroke
Source: Transl Stroke Res. 2021 Feb 24;12(6):991–1017. doi: 10.1007/s12975-021-00888-3 (PMC8557200; doi:10.1007/s12975-021-00888-3)
Supplement: Supplementary file 1 — (DOC 109 kb) [file 12975_2021_888_MOESM1_ESM.doc]

**Supplementary tables**

**Supplementary Table 1. Primary antibodies used in this study**

| **Protein** | **Usage** | | **Antibody** |
| --- | --- | --- | --- |
| GFAP | IF (1:500), IHC (1:500) | Abcam, ab7260 | |
| GFAP | IHC (1:1000), WB (1:5000) | Abcam, ab10062 | |
| Map-2 | IHC (1:500) | Abcam, ab32454 | |
| Neurocan | IHC (1:100), WB (1:500) | Abcam, ab26003 | |
| phosphacan | IHC (1:100), WB (1:500) | Sigma, P8874 | |
| VEGF-D | IHC (1:200), WB (1:2000) | Abcam, ab155288 | |
| VEGFR3 | IHC (1:50), WB (1:50) | Abcam, ab27278 | |
| RIP1K | WB (1:1000) | Sigma，SAB3500420 | |
| RIP3K | WB (1:1000), IHC (1:300) | Abcam, ab62344 | |
| MLKL | WB (1:1000), IHC (1:100) | Biorbyt, orb32399 | |
| β-actin | WB (1:5000) | Sigma, A5441 | |
| β-Ⅲ-tubulin | IF (1:500) | Abcam, ab78078 | |

Abbreviations: WB, Western blotting; IF, Immunofluorescence; IHC,Immunohistochemistry

**Supplementary Table 2. Secondary antibodies used in this study**

| **Protein** | **Usage** | **Antibody** | |
| --- | --- | --- | --- |
| Alexa Fluor® 594 goat anti-rabbit IgG (H+L) | IF (1:500),  IHC (1:500) | | A11012, lifetechnologies |
| Alexa Fluor® 594 goat anti-mouse IgG (H+L) | IF (1:500),  IHC (1:500) | | A11005, lifetechnologies |
| Alexa Fluor® 488 goat anti-rabbit IgG (H+L) | IF (1:500),  IHC (1:500) | | A11008, lifetechnologies |
| Alexa Fluor® 488 goat anti-mouse IgG (H+L) | IF (1:500),  IHC (1:500) | | A11001, lifetechnologies |
| Anti-mouse IgG (H+L) | WB (1:10000) | | 042-06-18-06, KPL |
| anti-rabbit IgG (H+L) | WB (1:10000) | | 042-06-15-06, KPL |

Abbreviations: WB, Western blotting; IF, Immunofluorescence,IHC,Immunohistochemistry

**Supplementary Table 3. Microarrays assay and data in this study**

| **List of down-regulated genes** | | | | | |
| --- | --- | --- | --- | --- | --- |
|  | **log2 (Ratio)** | **P-value (Differentially expressed)** |  | **log2 (Ratio)** | **P-value (Differentially expressed)** |
| **Gene_symbol** | **T/C** | **T/C** | **Gene_symbol** | **T/C** | **T/C** |
| Slc25a45 | -1.845791 | 1.78E-24 | Angpt1 | -1.718949 | 0.012392577 |
| Figf | -2.885578 | 6.8E-23 | Mylpf | -0.935802 | 0.012670508 |
| Hbb-b1|LOC100134871|Hbb | -5.258582 | 1.48E-20 | Stbd1 | -0.909061 | 0.015798677 |
| Ahr | -1.744865 | 6.25603E-11 | Rxrg | -0.784905 | 0.016340803 |
| Tbx15 | -1.767157 | 6.27576E-11 | Plscr4 | -0.732494 | 0.01658766 |
| Irf5 | -1.448049 | 1.30057E-08 | Skil | -0.630148 | 0.01881152 |
| Bmp2 | -1.516133 | 4.98656E-08 | Emilin2 | -0.721499 | 0.019405 |
| Grin3a | -1.068653 | 2.27282E-07 | Lima1 | -0.779693 | 0.019722726 |
| Gnl3l | -0.792522 | 5.21424E-06 | Apobec2 | -0.604109 | 0.019874923 |
| Srpk2 | -1.369071 | 7.55951E-06 | Car13 | -1.603883 | 0.020247523 |
| Ccdc125 | -1.370078 | 1.29454E-05 | Hs6st1 | -1.088415 | 0.020558829 |
| Appl1 | -0.910917 | 2.09655E-05 | Wdyhv1 | -0.770071 | 0.022609344 |
| Tnfsf15 | -1.103595 | 2.51181E-05 | Prkca | -0.648261 | 0.025593644 |
| Abcg3l3 | -1.186185 | 3.99059E-05 | Atp10a | -0.659209 | 0.027129758 |
| RGD1305254 | -0.90465 | 0.000169203 | Slitrk6 | -1.69479 | 0.028864229 |
| Anks1b | -1.777759 | 0.000330007 | Jag1 | -0.653846 | 0.030463871 |
| Myh3 | -0.967073 | 0.000331085 | Ankrd50 | -0.702023 | 0.031622708 |
| Tnfrsf9 | -0.868915 | 0.000649952 | Fmo5 | -1.102617 | 0.032026902 |
| Blvra | -0.697513 | 0.00190908 | Ankrd1 | -0.759184 | 0.032197993 |
| Chd1 | -0.850696 | 0.002913315 | Rgs10 | -0.602427 | 0.032397617 |
| Wfdc1 | -0.731113 | 0.003546219 | Ccdc23 | -0.599153 | 0.033316393 |
| Myom1 | -0.655843 | 0.003986279 | Lmbr1l | -0.670631 | 0.034985784 |
| Cpne7 | -0.684283 | 0.006082015 | Igfbp5 | -1.470558 | 0.038940705 |
| Oosp1-ps1 | -0.786599 | 0.00779568 | Las1l | -0.631036 | 0.040956736 |
| Adora2b | -0.772062 | 0.007971236 | Epas1 | -0.719327 | 0.04219006 |
| RGD1311756 | -0.851426 | 0.008898987 | Hscb | -0.854386 | 0.044146318 |
| Ccdc69 | -0.733366 | 0.00918841 | Sema4a | -0.630784 | 0.044854768 |
| Tnfrsf1b | -1.166746 | 0.009392433 | Igfbp3 | -0.608194 | 0.046268404 |
| Vof16 | -1.088539 | 0.009674049 | Endod1 | -1.369405 | 0.046869636 |
| Rasgrp3 | -0.961626 | 0.010363746 | Icmt | -0.60111 | 0.047760215 |
| Tnfaip8 | -1.196121 | 0.010388171 | Fam35a | -0.810765 | 0.048424054 |
| Tmem8c | -0.776341 | 0.012234129 |  |  |  |
| **List of up-regulated genes** | | | | | |
|  | **log2 (Ratio)** | **P-value (Differentially expressed)** |  | **log2 (Ratio)** | **P-value (Differentially expressed)** |
| **Gene_symbol** | **T/C** | **T/C** | **Gene_symbol** | **T/C** | **T/C** |
| Gnao1 | 0.755569 | 2.17194E-06 | LOC100909537 | 0.685433 | 0.010886907 |
| LOC363267 | 0.896643 | 1.10801E-05 | Fam196a | 0.626352 | 0.011178748 |
| Dnajb13 | 0.726726 | 8.73745E-05 | Agbl2 | 0.694158 | 0.013768354 |
| Vwa3b | 0.850765 | 0.000226332 | Fank1 | 0.922835 | 0.013827561 |
| RGD1310641 | 0.869383 | 0.000294784 | Tmem212 | 0.773909 | 0.016923271 |
| Ccdc153 | 1.12015 | 0.000458174 | Pla2g7 | 0.593674 | 0.017063068 |
| Tf | 0.843547 | 0.000582168 | Wdr38 | 0.647975 | 0.017197885 |
| Fam227a | 0.937883 | 0.000604975 | Sorl1 | 0.640665 | 0.017281875 |
| Dnaaf1 | 0.826192 | 0.000656446 | Scg3 | 0.738671 | 0.019330943 |
| LOC680693 | 0.864229 | 0.000773241 | Efhb | 0.629274 | 0.019598013 |
| RGD1560020_predicted | 1.004575 | 0.000868494 | Ccdc164 | 1.158939 | 0.021286264 |
| Spag6 | 0.898798 | 0.000940155 | Dnai1 | 0.85984 | 0.022095216 |
| LOC685158 | 0.853519 | 0.001344131 | Oasl2 | 0.828218 | 0.022928001 |
| Dnah12 | 1.1048 | 0.001713758 | Wdr38 | 0.706687 | 0.024047943 |
| RGD1560386 | 0.858659 | 0.001776283 | Fos | 0.810466 | 0.026139112 |
| RGD1560137 | 0.864243 | 0.002415024 | Tnfrsf19 | 0.773031 | 0.027023211 |
| RGD1562658 | 0.788108 | 0.002509276 | Lrrc46 | 0.670373 | 0.029468132 |
| Lrrc23|LOC100911585 | 0.681453 | 0.003037869 | Tppp3 | 1.316861 | 0.029707676 |
| RGD1559985 | 0.611747 | 0.003092939 | Rasd1 | 0.585514 | 0.029767931 |
| Gdf15 | 1.150437 | 0.003319446 | Fam183b | 1.418457 | 0.034256723 |
| Wdr63 | 0.929717 | 0.003539834 | Ccdc19 | 0.89704 | 0.035607405 |
| Rsph10b | 0.987434 | 0.003876255 | LOC100912469 | 0.686627 | 0.037044372 |
| Ppp1r32 | 0.922707 | 0.004218695 | RGD1561916 | 1.089067 | 0.037972767 |
| Ednrb | 1.236162 | 0.004338935 | Stpg1 | 0.947765 | 0.039419383 |
| Dmc1 | 0.723874 | 0.004944155 | Abhd3 | 0.745467 | 0.039599534 |
| Ccdc146 | 0.727664 | 0.005065292 | Spag8 | 0.760677 | 0.040358085 |
| RGD1564149 | 0.995941 | 0.005570074 | Cyp1a2 | 0.732916 | 0.041377954 |
| Metrn | 0.977188 | 0.006072478 | Folr1 | 0.925764 | 0.041631606 |
| Vcan | 0.619807 | 0.006519239 | Zmynd10 | 0.796458 | 0.042143922 |
| Sfxn5 | 0.779743 | 0.006864022 | Mfrp | 0.665477 | 0.042645425 |
| Rsph4a | 0.825832 | 0.007281841 | Riiad1 | 0.850951 | 0.044657316 |
| Hrk | 0.670482 | 0.008861701 | Mt1a | 0.747908 | 0.044926215 |
| RGD1565611 | 0.649988 | 0.009826548 | Dynlrb2 | 1.211319 | 0.047000974 |
| Usp43 | 1.044232 | 0.010019019 | Calml4 | 0.636316 | 0.047893047 |
| Vwa3a | 0.841388 | 0.010051361 | Dnali1 | 0.913268 | 0.047974136 |
| Msx3 | 0.855063 | 0.010244462 |  |  |  |
